# UMobileNetV2 model for semantic segmentation of gastrointestinal tract in MRI scans

**DOI:** 10.1371/journal.pone.0302880

**Published:** 2024-05-08

**Authors:** Neha Sharma, Sheifali Gupta, Deepali Gupta, Punit Gupta, Sapna Juneja, Asadullah Shah, Asadullah Shaikh

**Affiliations:** 1 Chitkara University Institute of Engineering and Technology, Chitkara University, Punjab, India; 2 University College Dublin, Dublin, Ireland; 3 Manipal University Jaipur, Jaipur, India; 4 International Islamic University, Kuala Lumpur, Malaysia; 5 Najran University, Najran, Saudi Arabia; Alexandria University Faculty of Nursing, EGYPT

## Abstract

Gastrointestinal (GI) cancer is leading general tumour in the Gastrointestinal tract, which is fourth significant reason of tumour death in men and women. The common cure for GI cancer is radiation treatment, which contains directing a high-energy X-ray beam onto the tumor while avoiding healthy organs. To provide high dosages of X-rays, a system needs for accurately segmenting the GI tract organs. The study presents a UMobileNetV2 model for semantic segmentation of small and large intestine and stomach in MRI images of the GI tract. The model uses MobileNetV2 as an encoder in the contraction path and UNet layers as a decoder in the expansion path. The UW-Madison database, which contains MRI scans from 85 patients and 38,496 images, is used for evaluation. This automated technology has the capability to enhance the pace of cancer therapy by aiding the radio oncologist in the process of segmenting the organs of the GI tract. The UMobileNetV2 model is compared to three transfer learning models: Xception, ResNet 101, and NASNet mobile, which are used as encoders in UNet architecture. The model is analyzed using three distinct optimizers, i.e., Adam, RMS, and SGD. The UMobileNetV2 model with the combination of Adam optimizer outperforms all other transfer learning models. It obtains a dice coefficient of 0.8984, an IoU of 0.8697, and a validation loss of 0.1310, proving its ability to reliably segment the stomach and intestines in MRI images of gastrointestinal cancer patients.

## 1. Introduction

The term "gastrointestinal tract" refers to humans and animals’ entire digestive system (from mouth to anus). The gastrointestinal (GI) tract, also known as the digestive tract, is a lengthy tubular organ that spans from the oral cavity to the rectum. Its primary function is to facilitate the breakdown and assimilation of nutrients from ingested food [1]. The gastrointestinal system is essential for the process of food digestion, where it breaks down food into smaller molecules that may be readily absorbed by the body, extracting nutrients for energy and growth, and eliminating waste products from the body [2]. It is also closely connected with the immune system and essential for maintaining overall health and well-being.

In the past 20 years, substantial advancements have been made to automatically diagnose the disorder in the digestive system and other human organs [1–5]. Gastrointestinal illness is a prevalent manifestation of these conditions [6]. Gastrointestinal cancer is the most prevalent kind of cancer in both males and females [5]. In 2019, the global incidence of gastrointestinal cancer exceeded 5 million cases. GLOBOCAN 2020 projections indicate that Gastrointestinal (GI) cancer claimed the lives of 800,000 individuals, accounting for 7.7% of all cancer-related deaths. It ranks as the fourth leading cause of cancer mortality in both men and women [7]. In the year 2020, a total of 1.1 million new instances of gastrointestinal (GI) cancer were identified, representing 5.6% of all cancer cases [8].

Treatment for gastrointestinal cancer is affected by the age of patients, their health, and the stage or type of cancer they suffer from [7]. The most common therapies for GI cancer are surgery, radiation treatment, and chemotherapy. Radiation treatment is usually given for 15 minutes daily for few weeks. Radiation oncologists employ this technique to treat cancers with solid doses of radiation while neglecting stomach and intestines [6]. With more recent technology like MRI and linear accelerator devices, commonly known as MR-Linacs, oncologists may see the tumor’s and intestines’ potential daily changes [8–10]. In order to provide large amounts of X-ray radiation, it is necessary for a system to precisely divide the organs in the gastrointestinal tract into distinct segments. This automated technology has the capability to enhance the pace of cancer therapy by aiding the radio oncologist in the process of segmenting the organs of the gastrointestinal tract.

Recent advancements show that deep learning algorithms are capable of segmenting GI tract organs [6–10]. Organ segmentation is essential for diagnostic and monitoring systems [11]. Deep learning algorithms, especially convolutional neural network-based architecture, are highly suitable to solve this issue of GI Tract segmentation [12]. Recent decades have seen encouraging results in Convolutional Neural Network, with the disorders diagnosing in various human organs. The CNN model is advantageous because it hierarchically extracts features, beginning with the most basic and working to the most abstract. The deep learning algorithms most efficiently used for model optimization are Dwarf Mongoose and Aquila Optimizer [13,14]. Clinical procedures such as diagnosis, therapy planning, and administration can benefit from organ segmentation. In this scenario, the digestive tract segmentation could benefit from a DL method, speeding up treatments and allowing patients to get more effective caution [12]. The proposed work has built a deep learning approach for automatic segmentation of the stomach and intestines in the Gastrointestinal tract in MRI scans. These MRI scans were taken during radiation treatment of actual cancer patients who experienced 1–6 scans per week, depending upon the stage of cancer. The main offerings of the anticipated research are as follows:

- Here, a UMobilenetV2 network is simulated by integrating MobileNet into the contraction path of UNet architecture. In contrast, layers of UNet are incorporated in the expansion path as the decoder to enhance the local feature extraction in the segmenting the GI tract using MRI images.
- The model has been implemented on the UW-Madison GI tract dataset to segment the stomach, small intestine, and large intestine in the GI tract. The model is examined using Adam, RMS prop, and SGD optimizers.
- The model is also compared with three transfer learning models named Xception, ResNet 101, and NASNet, which are used as encoders in UNet architecture. The approach is assessed based on performance parameters like model loss, dice coefficient, and IoU.

The leftover sections of this article are ordered as section 2 presents the related work for classification and segmentation in the GI tract. Section 3 describes the methodology for this research work. Section 4 shows results and discussion, section 5 shows state-of-the-art comparison, and section 6 concludes the overall job done in this research.

## 2. Related work

In recent years, several researchers have worked the categorization and segmentation of the gastrointestinal system. Table 1 summarises current, significant learning-based advancements in this domain. Cogan T. et al. [15] created the MAPGI framework in 2019 for modular and automated pre-processing of gastrointestinal images. For the Kvasir dataset, some pre-processing procedures include edge elimination, filtering, and color mapping. Deep learning architectures, Inception-v4, Inception-v2, and NASNet, achieved accuracy scores of 0.9845, 0.9848, and 0.9735 for GI Tract segmentation. Sharif M. et al. [16] proposed an approach to merging deep convolutional and geometric characteristics in 2019. The suggested technique was evaluated on a database of 5500 images and demonstrated classification accuracy and precision of 99.42% and 99.51%, respectively. Gamage C. et al. 2019 predicted eight-class GI disease anomalies using a mixture of DenseNet-201, VGG-16, ResNet-18, and CNN followed by a global average pooling layer [17]. D. E. Diamantis et al. proposed a strategy for coping with the inadequate data in 2019 by employing synthetically created pictures. A CNN was trained utilising WCE photographs [18]. Ozturk S. suggested an incredibly well-organized LSTM model, which will be merged into CNN output in 2020 [19]. Lafraxo S. et al. proposed a DL model which employs a deep convolutional network and achieves 96.89% accuracy on the Kvasir dataset [20]. Hmoud Al-Adhaileh, M. et al. used the Kvasir dataset to train GoogleNet, ResNet-50, and AlexNet deep learning-based networks in 2021. AlexNet provided the best results, with 97% accuracy [21]. Yogapriya J. et al. used classic image processing methods, a data augmentation strategy, and a deep network to categorize GI disorders in wireless endoscopic pictures [22]. In [23], S. Ozturk introduced a model that combines a CNN with a residual LSTM. Montalbo et al. [24] recommended the Multi-Fused Residual CNN (MFuRe-CNN) for analyzing endoscopic images of GI illnesses using the Kvasir dataset in 2022. Gibson et al. reported a neural network design for segmentation of eight organs [25]. The pancreas, digestive system, "esophagus, stomach, and duodenum," are all necessary for endoscopic referral to the biliary and pancreatic processes. Wang S. et al. published a multi-scale deep network in 2020 to eventually segment gastrointestinal (GI) lesions from endoscopic images [26]. Khan M. A. et al. proposed an approach for categorizing and diagnosing GI ulcers, polyps, and hemorrhages in 2020. It was recommended in [27] to employ a Recurrent CNN tailored for ulcer segmentation. Garden et al. [28] 2021 established a technique for segmentation of canonical method appropriate to identifying GI polyps via a direct extension [29]. According to the literature, the gastrointestinal system has been the substantially researched in last years, including classification and segmentation. The study made use of a variety of datasets, including endoscopic and CT scan pictures. The proposed study uses MRI images to provide a unique method for segmenting the stomach and intestines in the GI system.

**Table 1:** Literature review on gastrointestinal tract.

S. no.	Ref./ Year	Dataset Name	Number of images	Technique	Segmentation	Classification	Performance Parameters
1	[23]/ 2018	CT scan images	680	Registration-free deep learning-based segmentation	✓	✗	Dice score: 0.78
2	[15]/ 2019	Kvasir dataset	8000	NASNet, Inceptionv2, and Inception-v4	✗	✓	Accuracy, NASNet: 0.9845 Inception-v2: 0.9848, Inception-v4: 0.9735
3	[16]/ 2019	privately collected database of Wireless Capsule Endoscopy (WCE) images	5500	Fusion of CNN + geometric features + KNN	✗	✓	Accuracy: 99.42%
4	[17]/ 2019	KVASIR Dataset	8000	Ensemble learning	✗	✓	Accuracy: 97%
5	[18]/ 2019	KID dataset	955	GAN +CNN	✗	✓	Accuracy: 90.9%
6	[19]/ 2020	Kvasir dataset	8000	CNN + LSTM	✗	✓	Accuracy: 97.90%
7	[20]/ 2020	Kvasir dataset	8000	CNN	✗	✓	Accuracy: 96.89%
8	[26]/ 2020	EndoVis-Ab and CVC-ClinicDB datasets	1310	MFuRe-CNN	✓	✗	mIoU, EndoVis-Ab: 74% CVC-ClinicDB: 85%
9	[27]/ 2020	Private dataset of WCE video/ images	682	Mask RCNN+ Resnet 101+SVM	✓	✓	MOC -0.8807 and accuracy 99.13%
10	[21]/ 2021	Kvasir dataset	5000	GoogleNet, ResNet-50, and AlexNet	✗	✓	Accuracy-97%, Sensitivity-96.8%, Specificity- 99.20%, and AUC-99.98%.
11	[22]/ 2021	WCE images	6702	VGG16, GoogLeNet and ResNet-18, and convolutional neural network (CNN)	✗	✓	Accuracy VGG 16–96.33%
12	[23]/ 2021	Kvasir dataset	8000	CNN	✗	✓	Accuracy: 98.05%
13	[28]/ 2021	Endoscopy images	720	encoder-decoder networks for semantic segmentation	✓	✗	Accuracy: 98%
14	[29]/ 2021	“Kvasir-Instrument” dataset.	590	U-Net architecture	✓	✗	Dice: 0.9158 and Jaccard index: 0.8578
15	[24]/ 2022	Kvasir dataset	8000	Fusion of CNN layer	✗	✓	Accuracy: 97.25%

## 3. Proposed methodology

**Fig 1:**
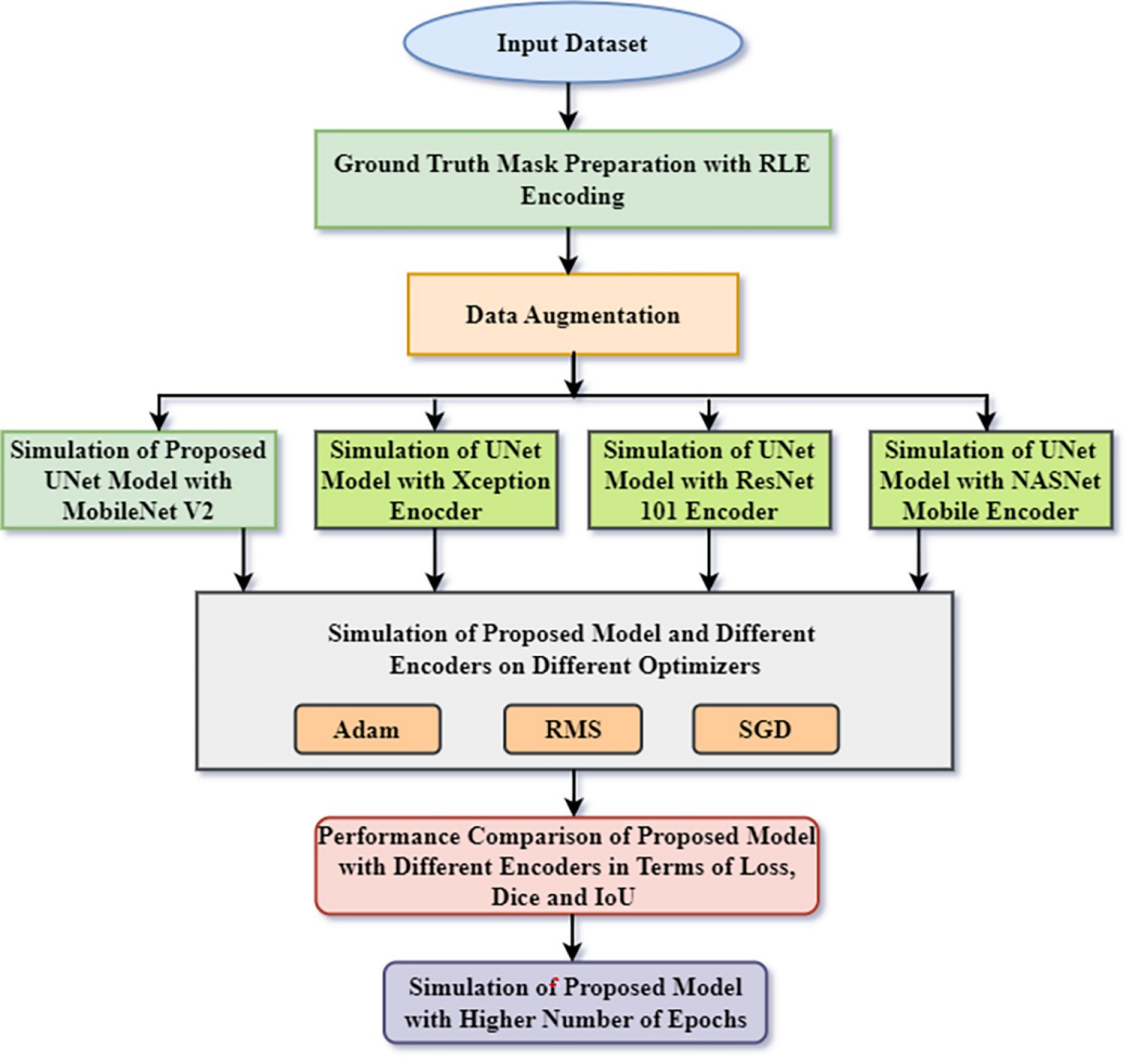
Flow chart for automatic segmentation of small bowel, large bowel, and stomach in GI tract.

This part discusses the methodology for segmentation and classification of the stomach, small bowel, and large bowel in MRI scans. Section A represents the input dataset. Section B represents the ground truth mask generation using Run Length Encoding (RLE). Section C will discuss the data augmentation applied to the dataset. Section D will discuss the model for segmenting the gastrointestinal tract. Section E shows the details of the three encoders used for UNet model. Section F shows the performance matrices used to analyze the model and three encoders. Fig 1 represents the flow chart of methodology for segmenting the stomach and intestines in the GI tract.

The Fig 1 displays the flow chart of the suggested technique. Fig 1 outlines a comprehensive process for semantic segmentation of MRI scans, utilizing UW Madison dataset comprising of 38,496 MRI images. The primary goal is to accurately segment GI tract organs such as small bowel, large bowel, and stomach from the input dataset. Beginning with the dataset input, ground truth masks are generated through Run-Length Encoding (RLE), paving the way for subsequent steps. Employing data augmentation techniques enhances model robustness. Here a semantic segmentation UMobileNet V2 model is simulated in which MobileNet V2 is used as an encoder in UNet Model for segmenting GI organs. The crux of the workflow lies in comparing the UMobileNet V2 model with three distinct encoders (Xception, ResNet 101, and NasNet Mobile). These models undergo optimization with three optimizers—Adam, RMSprop, and SGD. Rigorous performance evaluation, utilizing metrics like loss, Dice coefficient, and Intersection over Union, facilitates comparison of model effectiveness. The proposed technique is further simulated with higher number of epochs to check its performance. The workflow concludes with the visualization of results from the best-performing model, offering a clear representation of the model’s prowess in accurately segmenting gastrointestinal structures within MRI scans. Overall, this systematic approach thoroughly explores segmentation methodologies, leading to informed model selection and meaningful insights into MRI image analysis.

### A. Input dataset

**Fig 2:**
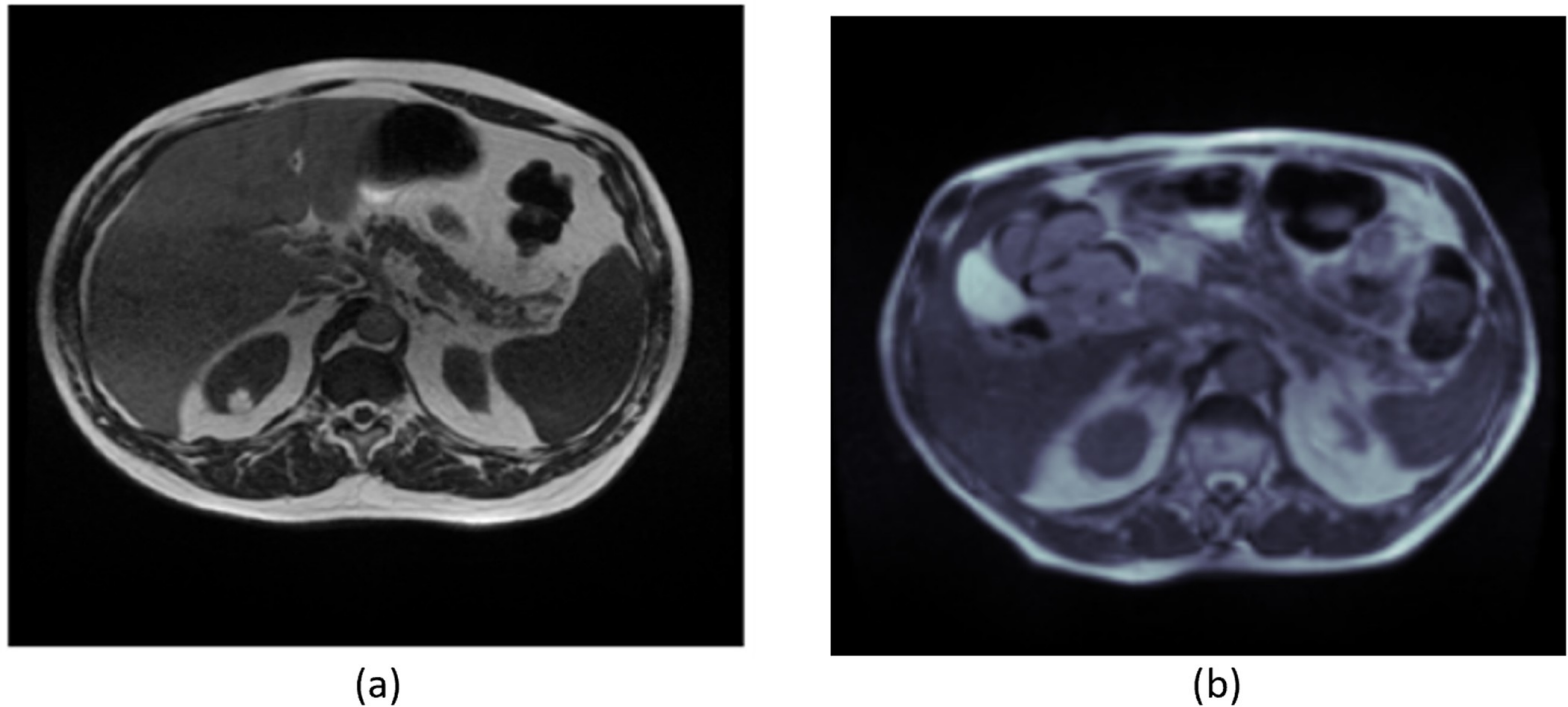
Images of the UW-Madison database.

The University of Wisconsin-Madison, a public research university in Madison, Wisconsin, has published a dataset of MRI scans [30]. The dataset comprises 85 individuals who underwent scans during a period ranging from 1 to 6 days. Each daily scan consists of either 144 or 80 slices, which are used for various patients. Therefore, the dataset has a total of 38496 MRI images. The images in the database vary, with sizes of 266x266, 310x360, and 276x276. All images were resized to 224x224 to make them uniform for training purposes. Fig 2A and 2B show sample MRI scans of database.

### B. Ground truth mask generation

**Fig 3:**
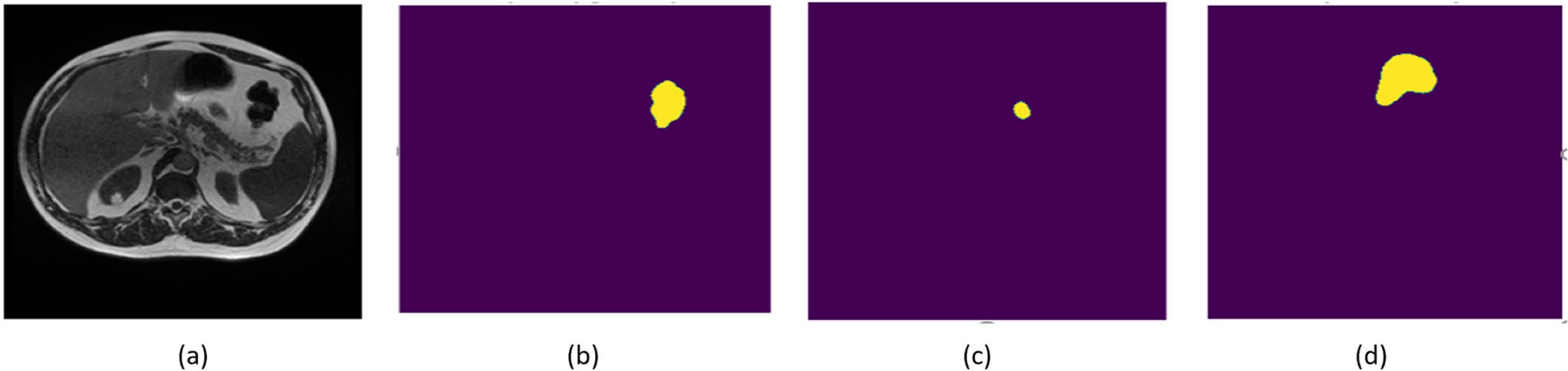
Ground Truth Mask Generation (a) Original Image, (b) RLE Encoding for Large Bowel, (c) RLE Encoding for Small Bowel, and (d) RLE Encoding for Stomach.

The dataset contains 38496 MRI slices, and each MRI slice has three annotations for Small bowel, large bowel, and stomach in RLE encoded forms provided in the CSV file. Hence, there are 115488 annotations given in the CSV file. Out of 115488 annotations, 14085 cases are for large bowel, 11201 are for small bowel, whereas 8627 cases are for stomach. The remaining 81575 annotation cases do not have any large, small, or stomachs. The ground truth mask is derived from these annotations using the RLE encoder. For example, Fig 3A shows the original 82-number slice of the 20th day’s scan of patient ID 123. Fig 3B shows the RLE encoding of the large intestine, Fig 3C represents the RLE encoding of the small intestine, and Fig 3D represents the RLE encoding of stomach of the same slice.

The Table 2 provides a breakdown of annotations for different anatomical regions, namely Large Bowel, Small Bowel, Stomach, and a category labeled as Blanks. The dataset is partitioned into training (80%), testing (10%), and validation (10%) subsets. In the training set, there are 11,989 annotations for the Large intestine, 8,961 for the Small intestine, 6,903 for the Stomach, and 65,261 for the Blanks category. The testing and validation sets each contain 1,408 annotations for the Large intestine, 1,120 for the Small intestine, 862 for the Stomach, and 8,157 for the Blanks category. These annotations likely represent a dataset used for training and evaluating proposed technique, for segmentation of GI tract organs.

**Table 2:** Dataset splitting in training, testing and validation.

	Large Bowel	Small Bowel	Stomach	Blanks
**Annotations**	14085	11201	8627	81575
**Training annotations**	11989	8961	6903	65261
**Testing annotations**	1408	1120	862	8157
**Validation annotations**	1408	1120	862	8157

### C. Data augmentation

**Fig 4:**
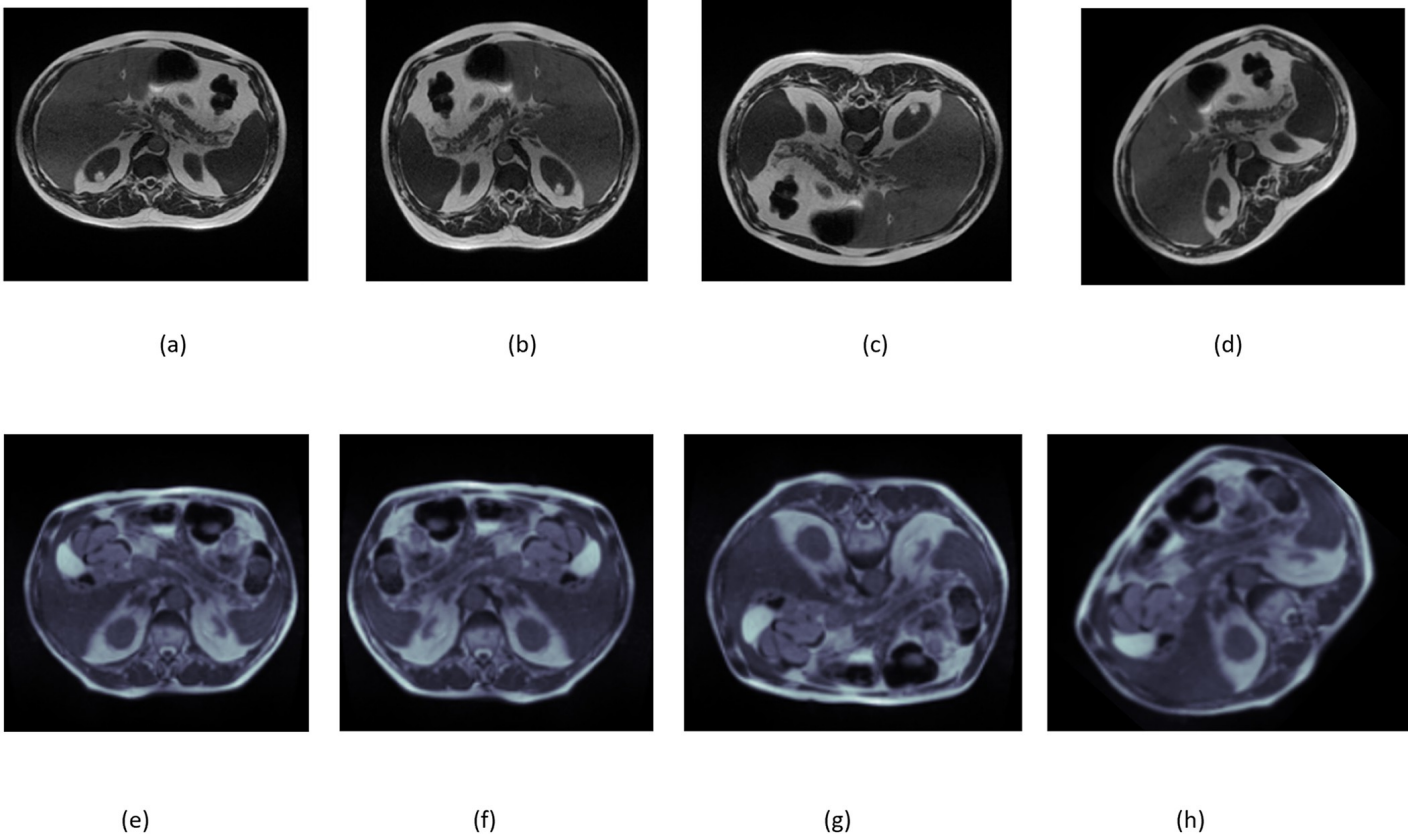
Sample Images After Applying the Augmentation Techniques; (a) & (e) Original Images, (b) &(f) Horizontal Flip, (c) & (g) Vertical Flip, and (d) & (h) Rotation.

The dataset is unbalanced here, with 14085 large bowel cases, 11,201 small cases, and 8,627 stomach cases. The dataset balancing is done on stomach cases using data replication by increasing its number from 8627 to 10783. Data augmentation is also applied to enhance the data to make it more compatible with the model. It enhances the diversity of images and acts as a dataset regularizer. It enhances the images by making alterations while preserving the class label. The augmentations employed in this dataset include horizontal flipping, vertical flipping, and rotation by 80° degrees. Fig 4 displays the unaltered and enhanced images derived from the dataset. Fig 4A and 4E display the original photos, (b) and (f) show the images after a horizontal flip, (c) and (g) show the images after a vertical flip, and (d) and (h) show the images after a rotation.

### D. UMobileNetV2 model

**Fig 5:**
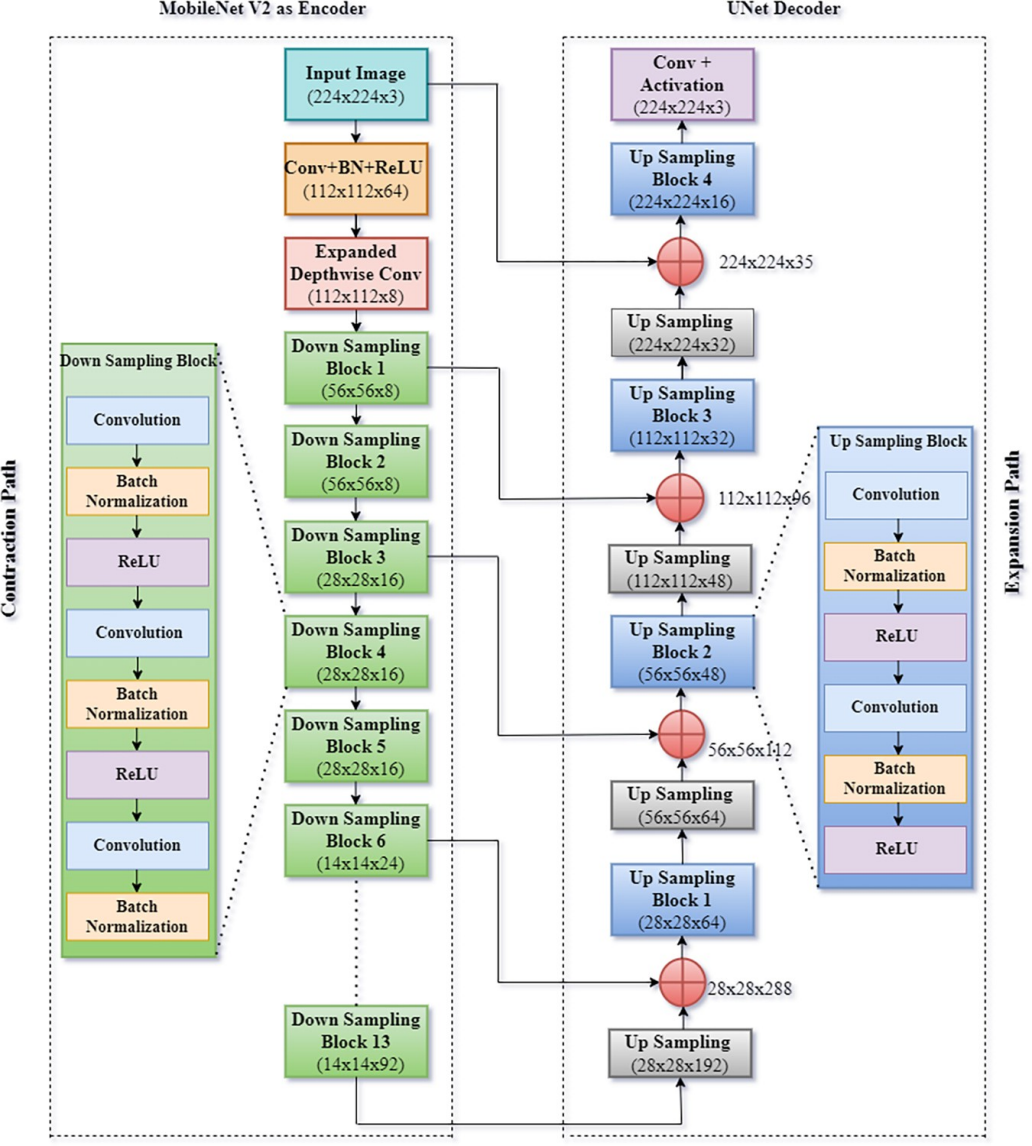
Block diagram of UMobileNetV2 for segmentation.

The simulated model is a fusion of UNet and mobilenet V2 model [31] for semantic segmentation of the large intestine, small intestine, and stomach in the GI tract on MRI data for cancer treatment. U-Net is a CNN model developed by Olaf Ronneberger et al. [32,33] for segmentation. U-Nets allow us to go beyond traditional image categorization and object recognition methods by assigning shapes to each pixel inside an image. It extends the conventional CNN architecture by adding a suitable expansion path (decoder) to provide a high-definition semantic prediction. Fig 5 shows a block schematic of the model. The first path is the encoder’s contraction path, which records the image’s features. The contraction is a structure comprised convolution and max pooling layers. Similarly, the expansion path (decoder) facilitates accurate localization by employing transposed convolutional layers; it does not include a dense layer and can process images of any shape. U-Net developed its name because its two branches resemble the letter U from the English alphabet.

Instead of a CNN, the proposed model used the transfer learning model MobileNet V2, which had already been pre-trained. MobileNet V2 [34] is a CNN design intending to improve performance on mobile devices. It is predicated on a backward residual structure, with the bottleneck levels connecting via residual nodes. Lightweight depthwise convolutions filter features are used as a non-linear source in the intermediate expansion layer. The convolutions in MobileNet V2 are separated depthwise. It reduces the number of parameters compared to a network constructed using ordinary convolutions.

Consequently, compact deep neural networks are generated. In place of a one 3x3 convolution layer followed by batch normalization and ReLU, MobileNet design uses a 2x2 convolution layer followed by batch normalization. Specifically, MobileNet V2 performed a 3x3 depthwise convolution and a 1x1 pointwise convolution. 13 downsampling blocks are used, each with its unique configuration of convolution, batch normalization, and ReLU layer. When an image is divided into blocks, it loses resolution but gains depth by increasing the feature maps. It is chosen for downsampling purposes in the proposed U-Net architecture because of its many benefits, such as its small size and low processing time. Table 3 shows a detailed description of the recommended model’s different layers.

**Table 3:** Detailed description of layers of the UMobileNet model.

S. No.	Layers	Input Image	Filter Size	Activation Function	Output Image Size	Number of Parameters
1	Input Image	224x224x3	-	-	224x224x3	0
2	Conv 1	224x224x3	3x3	ReLU	112x112x64	496
3	expanded_conv_depthwise	112x112x64	3x3	ReLU	112x112x8	368
4	Down_sampling_block 1	112x112x8	3x3	ReLU	56x56x8	1616
5	Down_sampling_block 2	56x56x8	3x3	ReLU	56x56x8	1616
6	Down_sampling_block 3	56x56x8	3x3	ReLU	28x28x16	2032
7	Down_sampling_block 4	28x28x16	3x3	ReLU	28x28x16	4768
8	Down_sampling_block 5	28x28x16	3x3	ReLU	28x28x16	4768
9	Down_sampling_block 6	28x28x16	3x3	ReLU	14x14x24	5568
10	Down_sampling_block 7	14x14x24	3x3	ReLU	14x14x24	9456
11	Down_sampling_block 8	14x14x24	3x3	ReLU	14x14x24	9456
12	Down_sampling_block 9	14x14x24	3x3	ReLU	14x14x24	9456
13	Down_sampling_block 10	14x14x24	3x3	ReLU	14x14x32	10640
14	Down_sampling_block 11	14x14x32	3x3	ReLU	14x14x32	15680
15	Down_sampling_block 12	14x14x32	3x3	ReLU	14x14x32	15680
16	Down_sampling_block 13	14x14x32	3x3	ReLU	14x14x92	6912
17	Up_sampling_layer 1	14x14x92	2x2	-	28x28x192	0
18	Concatenate1	28x28x192	-	-	28x28x288	0
19	Up_sampling_block 1	28x28x288	3x3	ReLU	28x28x64	203392
20	Up_sampling_layer 2	28x28x64	2x2	-	56x56x64	0
21	Concatenate 2	6x56x64	-	-	56x56x112	0
22	Up_sampling_block 2	56x56x112	3x3	ReLU	56x56x48	69600
23	Up_sampling_layer 3	56x56x48	2x2	-	112x112x48	0
24	Concatenate 3	112x112x48	-	-	112x112x96	0
25	Up_sampling_block 3	112x112x96	3x3	ReLU	112x112x32	37184
26	Up_sampling_layer 4	112x112x32	2x2	-	224x224x32	0
27	Concatenate 4	224x224x32	-	-	224x224x35	0
28	Up_sampling_block 4	224x224x35	3x3	ReLU	224x224x16	7504
29	Conv	224x224x16	1x1	ReLU	224x224x3	51

Total Parameters: 416,243.

Trainable Parameters: 409,059.

Non-trainable Parameters: 7,184.

The computation cost of the UMobileNet V2 model is also measured in form of FLOPs that involves multiplying the number of operations per parameter by the total trainable parameters, the batch size, and the number of training iterations. The formula for FLOPs can be expressed as:

FLOPs = 2×Number of Operations per Parameter×Trainable Parameters×Batch Size×Number of Iterations

Here number of operations per parameter is assumed to be 2, Trainable Parameters are equal to 409,059 and Batch Size is taken as 16. So number of FLOPs used in the UMobileNet V2 model is 2×2×409,059×16×1 = 13,097,152.

So, for one iteration with a batch size of 16, the computation cost is approximately 13,097,152 FLOPs. The computational cost of the proposed model is comparatively less than other encoders because here MobileNet V2 is used as encoder. MobileNetV2 employs depthwise separable convolutions, a technique that divides a conventional convolution into two distinct operations: a depthwise convolution and a pointwise convolution. This minimises the quantity of parameters and calculations compared to traditional convolutions, leading to lower computational cost.

### E. Simulations parameters

In addition to the model’s structure, it is also essential to recognize the network’s execution and presentation. During the deep neural model’s training, many parameter choices were made. The MobileNet V2 model was used to build the proposed network, which was compared with three different transfer learning methods namely Xception, ResNet 101, and NASNet mobile. The model’s weights were set through Golort initialization [35]. The loss function used for the simulation is Tversky loss. It is commonly used as a loss function in image segmentation tasks, especially in medical image analysis. The formula for calculating Tversky loss is:

$$Tversky\;Loss=1-\frac{TP}{TP+\alpha FP+\beta FN}$$

Where TP is true positive, FP is a false positive, FN is false negative, α and β are weight parameters that allow adjusting the balance between false positive and false negative.

The model’s performance has been evaluated using several parameters: Adam, RMS, and SGD. Batch sizes of 16 and 10 epochs were used to run the model. These parameters were assessed using UW Madison dataset. The model’s learning rate is 0.0001. Python and KerasTensorflow [36] Package were used to build model. Keras is a free and simple tool for developing neural networks. NVIDIA Tesla P100 GPU is used for the simulation. It is open-source and compatible with Tensorflow and Theano. All the simulations were carried out using google colab notebook.

### F. Different encoders used for UNet model

Transfer learning is a method that reuses a network proficient for a job as an initial step of a model for a second relevant job. The idea is to transfer knowledge gained from solving one problem to another related issue so that less data can be used to train a more accurate or efficient model. This is especially useful when labelled data for new tasks is scarce.

#### a.  Xception model

Xception [37] is a deep network aimed to overcome the limitations of starting models for image classification tasks. Xception uses depth-separable convolutions, which can significantly reduce computational complexity and improve model performance. This architecture allows models to learn more efficiently by reducing the parameters while preserving solid, expressive power. Xception models are used for various computer vision tasks like recognition, segmentation, and fine-grained picture categorization.

#### b.  ResNet 101 model

ResNet-101 is a deep CNN competent in accomplishing image categorization tasks. Featured in his 2016 article "Deep Residual Learning for Image Recognition" by Microsoft researchers He, Zhang, Ren, and Sun [38]. This model is an extension of the ResNet 50 model, a variation of the traditional CNN architecture. The core concept behind the ResNet network is the introduction of residual connections that allow the network to learn its ID function in addition to the traditional convolution and pooling layers. The number "101" in the model name denotes the layers in the model, which are much more profound than other CNN architectures, such as VGG-16 and AlexNet. As a result, ResNet-101 can learn more powerful and complex feature representations from the input data, improving the performance of image classification tasks.

#### c.  NASNet model

NASNet (Neural Architecture Search Network) Mobile is a deep CNN developed for picture identification tasks and designed to be implemented on mobile and implanted strategies with limited computational resources. The model was introduced in the paper "Learning Transferable Architectures for Scalable Image Recognition" by Google researchers Zoph and Le in 2017 [39]. NASNet is based on the Automatic Neural Architecture (NAS) search method, which automatically uses reinforcement learning to find the optimal network architecture. This method learns to identify the best building blocks for your model and its placement.

### G. Performance metrics

Intersection over union (IoU) and Dice Coefficient are often employed metrics for evaluating the efficacy of segmentation methods.

*a*. *IoU*: The Jaccard index is another name for it. This is one of the most commonly used metrics for segmentation.

$$IoU=\frac{area\;of\;overlap}{area\;of\;union}$$

The Intersection over Union (IoU) is calculated by dividing the region of overlap between the expected and real segmentation by the area of union between the anticipated and actual segmentation. The measurements span a scale of 0 to 1, where a value of 0 indicates no overlap and a value of 1 indicates perfect overlap.

*b*. *Dice*: The term "F1 score" is also used to refer to it. The dice coefficient is calculated by multiplying the area of overlap between two images by two, and then dividing it by the total number of pixels in both images.

$$Dice\;Coefficent=\frac{2*area\;of\;overlap}{total\;number\;of\;pixels\;in\;both\;images}$$

There is a positive correlation between the Dice coefficient and the IoU coefficient. Both ranges span from 0 to 1, where a value of 1 indicates the highest degree of similarity between the predicted and actual outcomes, while a value of 0 indicates the lowest level of resemblance.

## 4. Results & discussions

The following sections show the results of the UMobileNet V2 model and UNet model simulated with three encoders with three different optimizers for segmentation of GI tract. The results were obtained using four encoders namely; MobileNet V2, Xception, ResNet 101, NASNet Mobile with three optimizers: Adam [40], RMS [41], and SGD [42].

### A.  Results for adam optimizer

This section shows the results of different encoders obtained using Adam optimizer.

#### a.  Loss analysis

**Fig 6:**
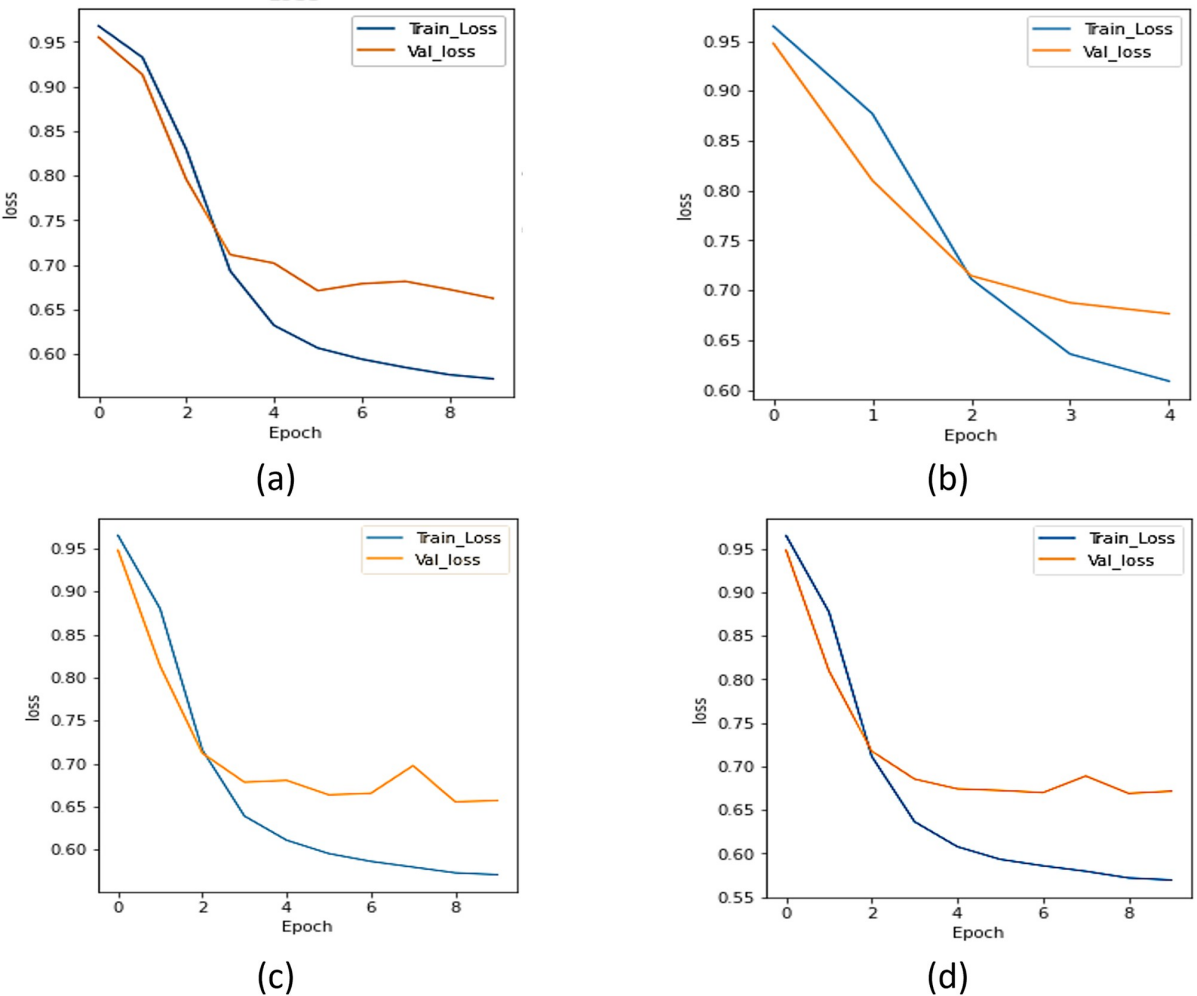
Loss analysis for different encoders using adam optimizer. (a) Xception, (b) ResNet 101, (c) NASNet Mobile, and (d) UMobileNet V2 Model.

The UNet model ensembles with different encoders were assessed using loss, dice, and IoU. Fig 6A displays the loss plot of the xception network, Fig 6B represents the loss plot for ResNet 101 model, Fig 6C displays the loss curve of the NASNet network, and Fig 6D represents the results of UMobileNet V2 model using Adam optimizer. From Fig 6, concludes that the UMobileNet V2 model obtains the least loss in comparison with other encoder networks.

#### b.  Dice coefficient analysis

**Fig 7:**
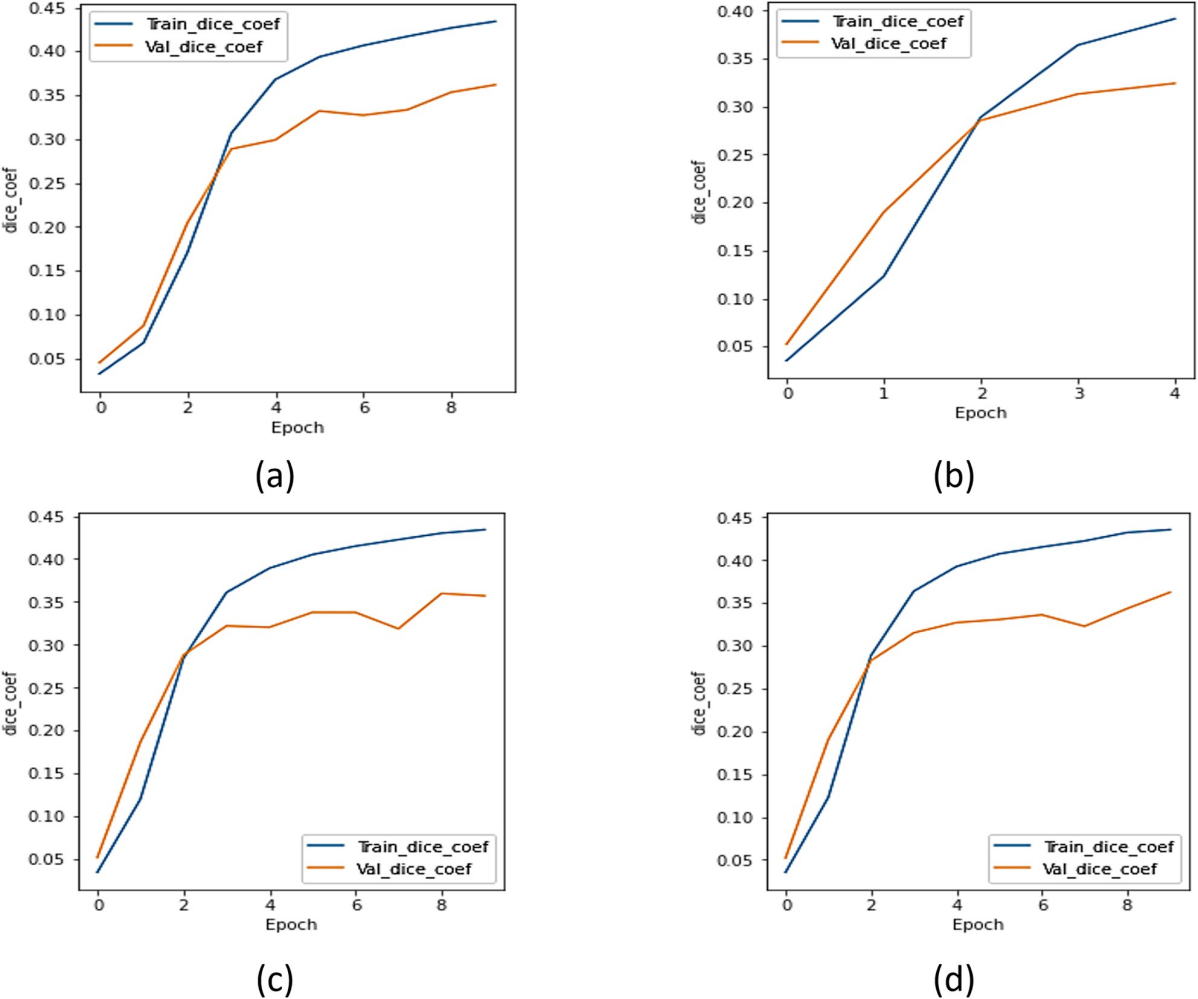
Dice coefficient analysis for different encoders using adam optimizer. (a) Xception, (b) ResNet 101, (c) NASNet Mobile, and (d) UMobileNet V2 Model.

The UNet with transfer learning designs were assessed utilizing the dice. Fig 7A represents the dice curve of xception model, Fig 7B represents the dice coefficient plot for ResNet 101 model, Fig 7C represents the dice coefficient curve of the NASNet model, and Fig 7D represents the results of UMobileNet V2 model using Adam optimizer. Fig 7 demonstrates that, compared to other models, the UMobileNet V2 model yields the most excellent dice coefficient value.

#### c.  IoU analysis

**Fig 8:**
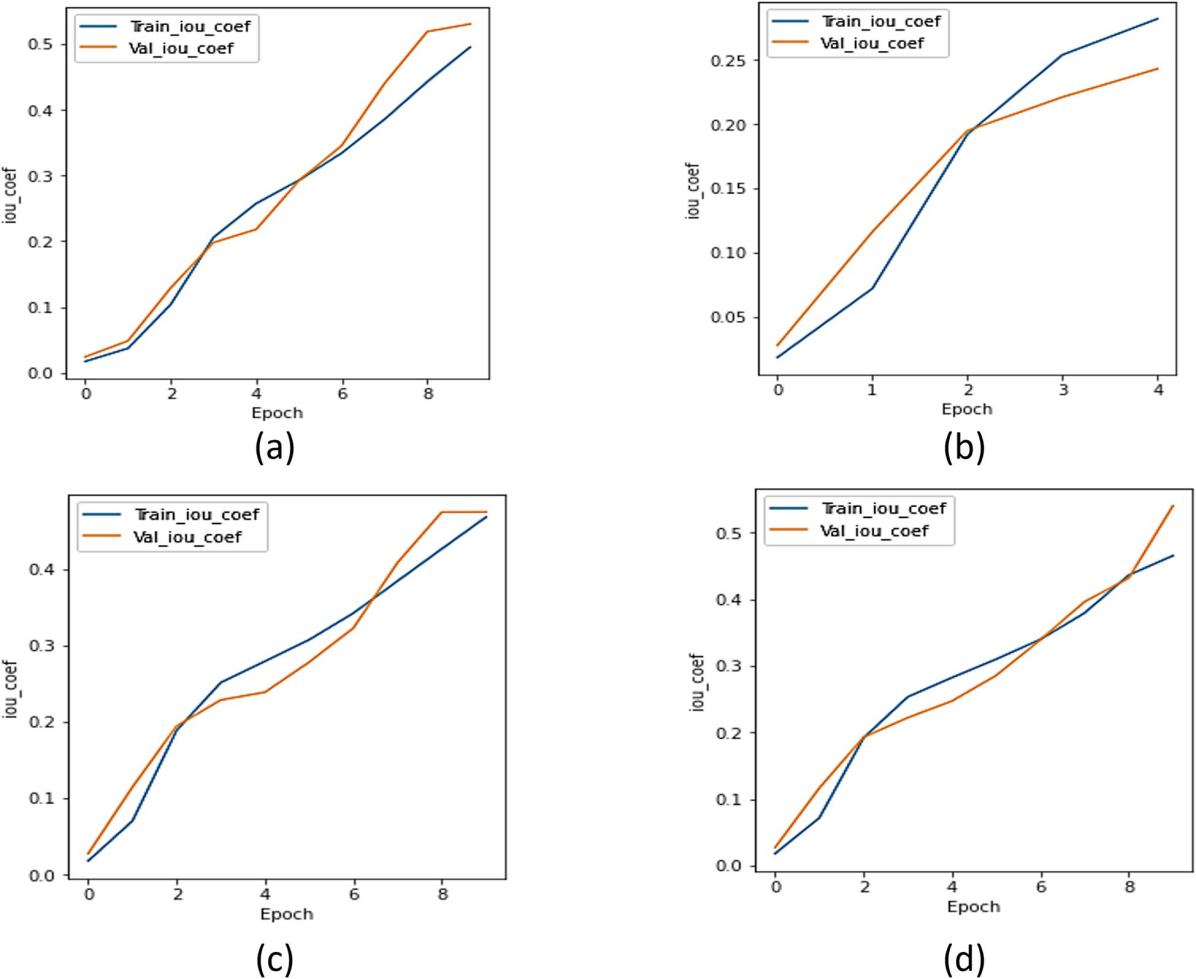
IoU Analysis for Different Encoders using Adam Optimizer (a) Xception, (b) ResNet 101, (c) NASNet Mobile, and (d) UMobileNet V2 Model.

The IoU coefficient was utilized to compare the UMobileNet V2 model to all other encoder models. Fig 8A displays the IoU curve for the xception model, Fig 8B shows the IoU curve for the ResNet 101 model, Fig 8C depicts the IoU curve for the NASNet model, and Fig 8D displays the plot of the model using the Adam optimizer. Regarding the IoU coefficient, Fig 8 concludes that the model performs better than every other transfer learning model.

**Fig 9:**
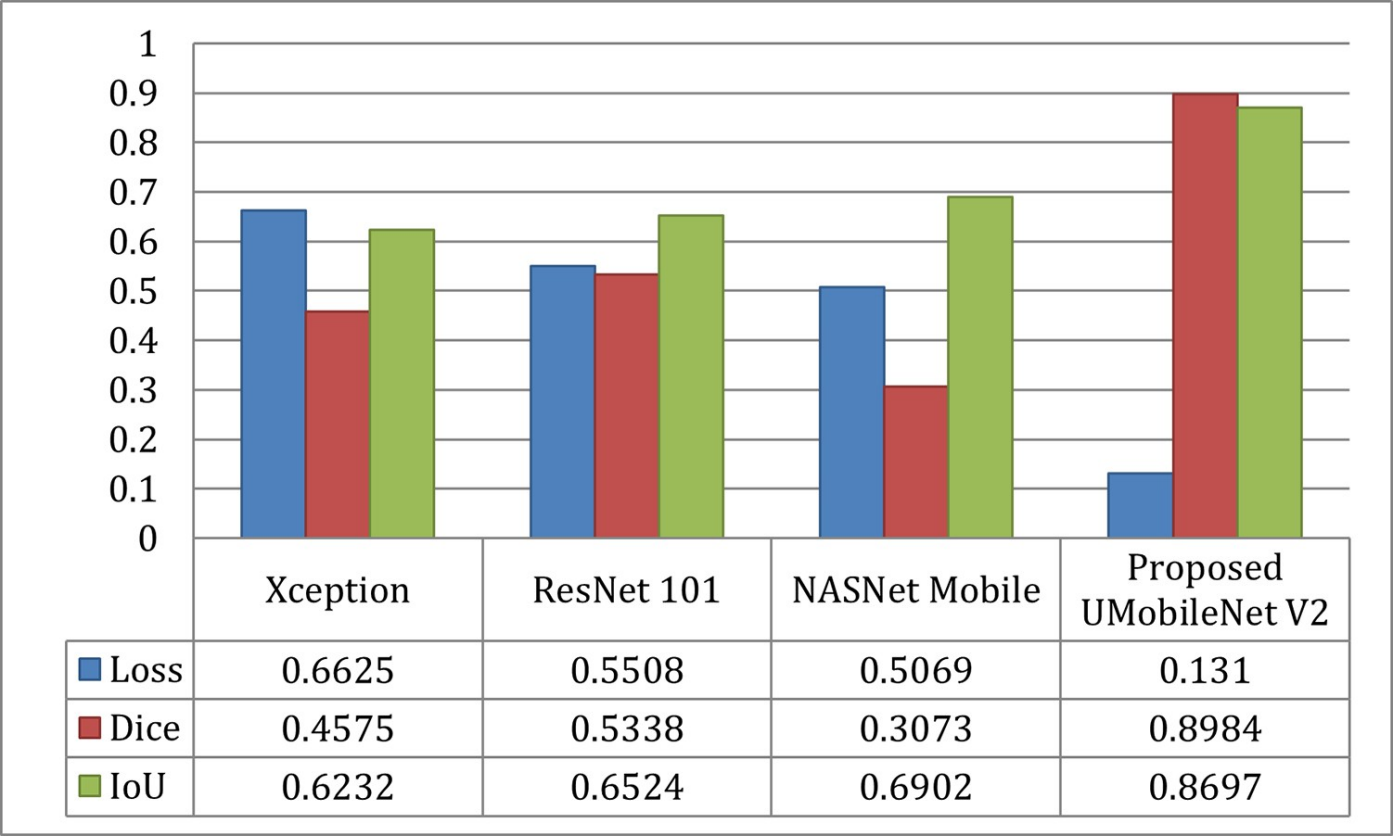
Results comparison of UMobileNet V2 model with different TL models with adam optimizer using test dataset.

Fig 9 compares the outcomes for the Adam optimizer for each model in terms of loss, dice coefficient, and IoU. The image shows that the UMobileNet V2 model performed better than previous transfer learning models. Using the Adam optimizer, the presented model produced the most significant dice coefficient with a value of 0.8904, the lowest loss value of 0.1310, and the best IoU value of 0.8697.

### B.  Results for RMS optimizer

The models’ loss, dice, and IoU are also evaluated using RMS optimizers. The following section shows the loss, dice, and IoU plots for different models using the RMS optimizer.

#### a.  Loss analysis

**Fig 10:**
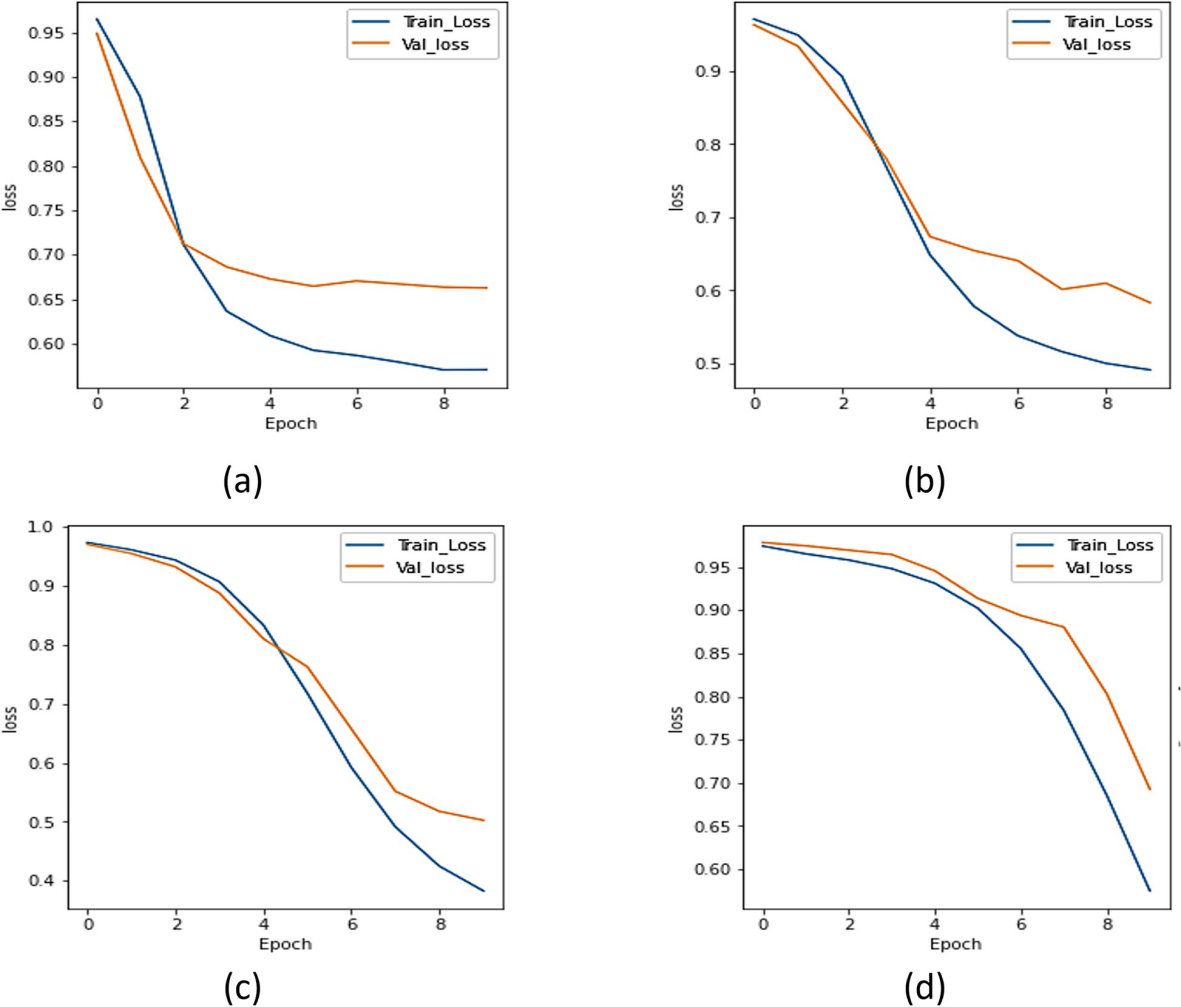
Loss Analysis for Different Encoders using RMS Optimizer (a) Xception, (b) ResNet 101, (c) NASNet Mobile, and (d) UMobileNet V2 Model.

To compare the ensemble UMobileNet V2 model with other transfer learning models, loss, dice coefficient, and IoU coefficient were considered. Fig 10A depicts the xception model’s loss curve, Fig 10B the ResNet 101 model’s loss curve, Fig 10C the NASNet model’s loss curve, and Fig 10D the UMobileNet V2 model’s RMS optimizer findings. Fig 10 demonstrates that the UMobileNet V2 model has the lowest loss value and a smoother slope than other models.

#### b.  Dice analysis

**Fig 11:**
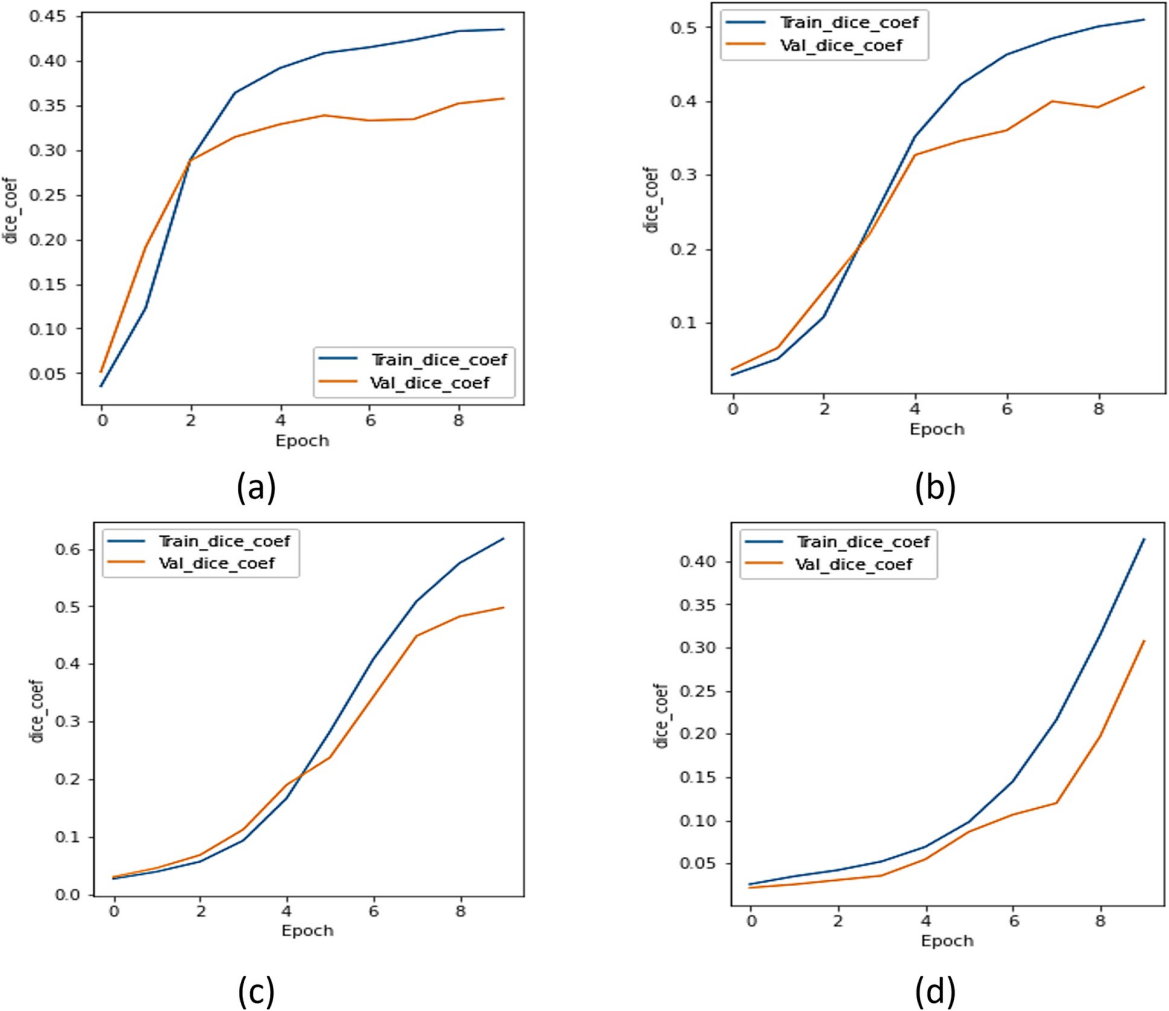
Dice coefficient analysis for different encoders using RMS optimizer. (a) Xception, (b) ResNet 101, (c) NASNet Mobile, and (d) UMobileNet V2 Model.

Fig 11A depicts the dice coefficient curve for the xception model, Fig 11B the dice coefficient curve for the ResNet 101 model, Fig 11C the NASNet model, and Fig 11D the outcomes of the UMobileNet V2 model employing RMS optimizer. Fig 11 demonstrates that the approach provides the maximum value for the dice coefficient. The presented model, which uses an RMS optimizer, may be said to beat any transfer learning model.

#### c.  IoU analysis

**Fig 12:**
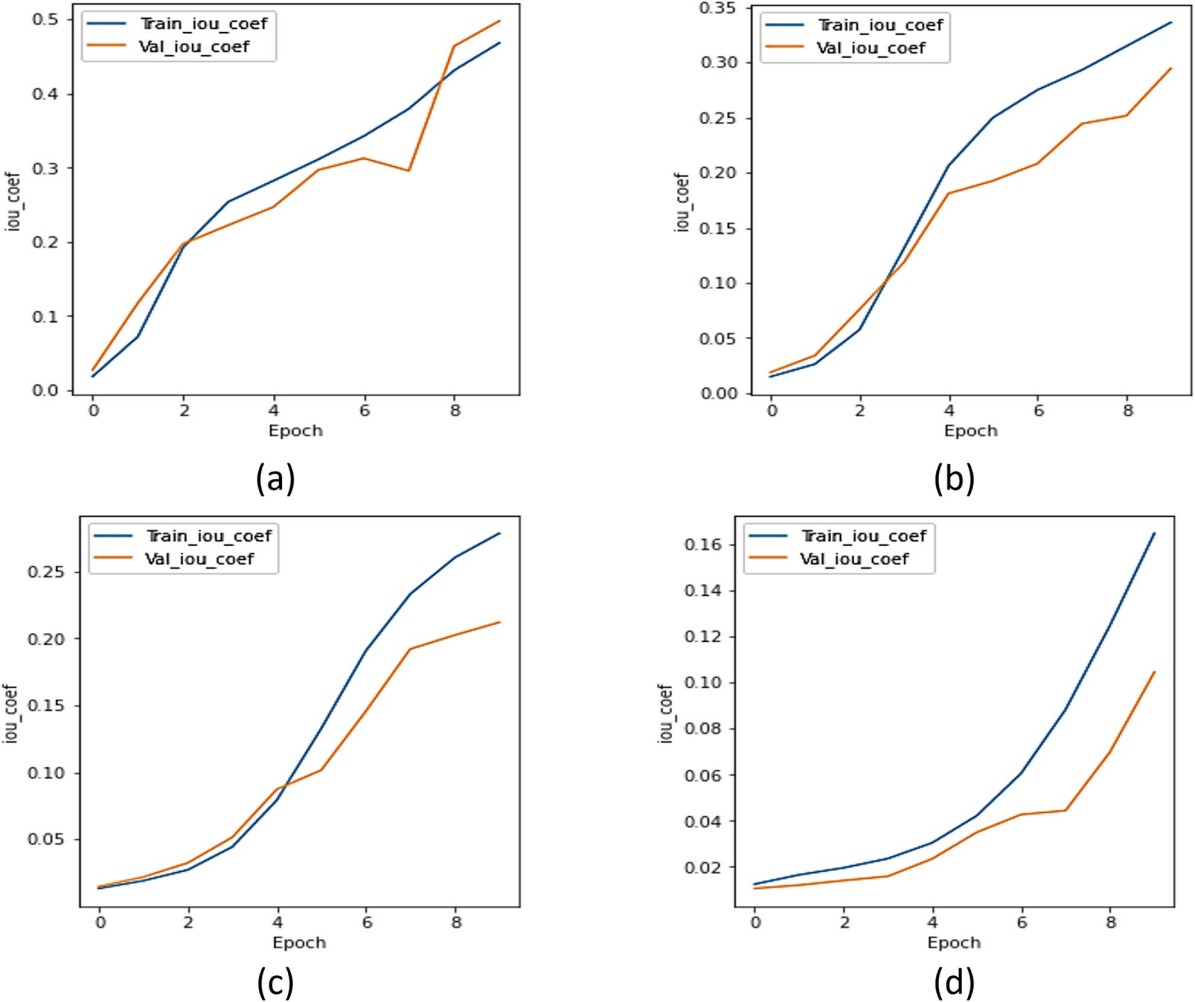
IoU analysis for different encoders using RMS optimizer. (a) Xception, (b) ResNet 101, (c) NASNet Mobile, and (d) UMobileNet V2 Model.

The IoU coefficient was utilised to compare the UMobileNet V2 model to all other encoder models. Fig 12A depicts the IoU curve for the xception model, Fig 12B the IoU curve for the ResNet 101 model, Fig 12C the IoU curve for the NASNet, and Fig 12D the plot of UMobileNet V2 model employing the RMS optimizer. Fig 12 demonstrates that the UMobileNet V2 model, which uses RMS optimization, has the greatest IoU value compared to other transfer learning models.

**Fig 13:**
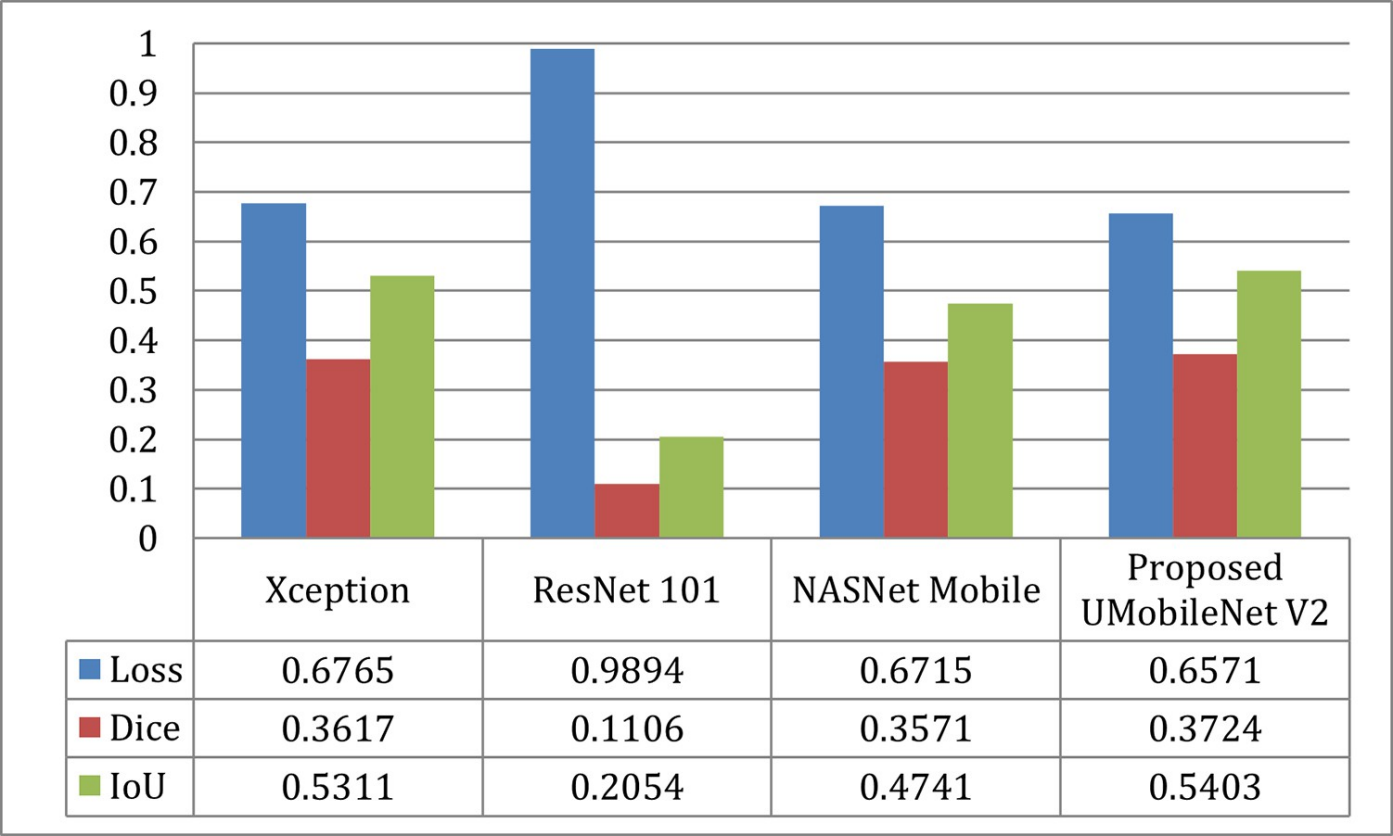
Results comparison of UMobileNet V2 model with different TL models with RMS optimizer using test dataset.

Fig 13 compares the RMS optimizer results for all models regarding loss, dice, and IoU. The UMobileNet V2 model achieves lowest loss, as can be deduced from Fig 13. The presented model uses an RMS optimizer to attain the most incredible value of the dice coefficient and IoU.

### C.  Results for SGD optimizer

The loss, dice, and IoU are evaluated using SGD optimizer for all models. The following sections show the loss, dice, and IoU plots for different models using SGD optimizer.

#### a.  Loss analysis

**Fig 14:**
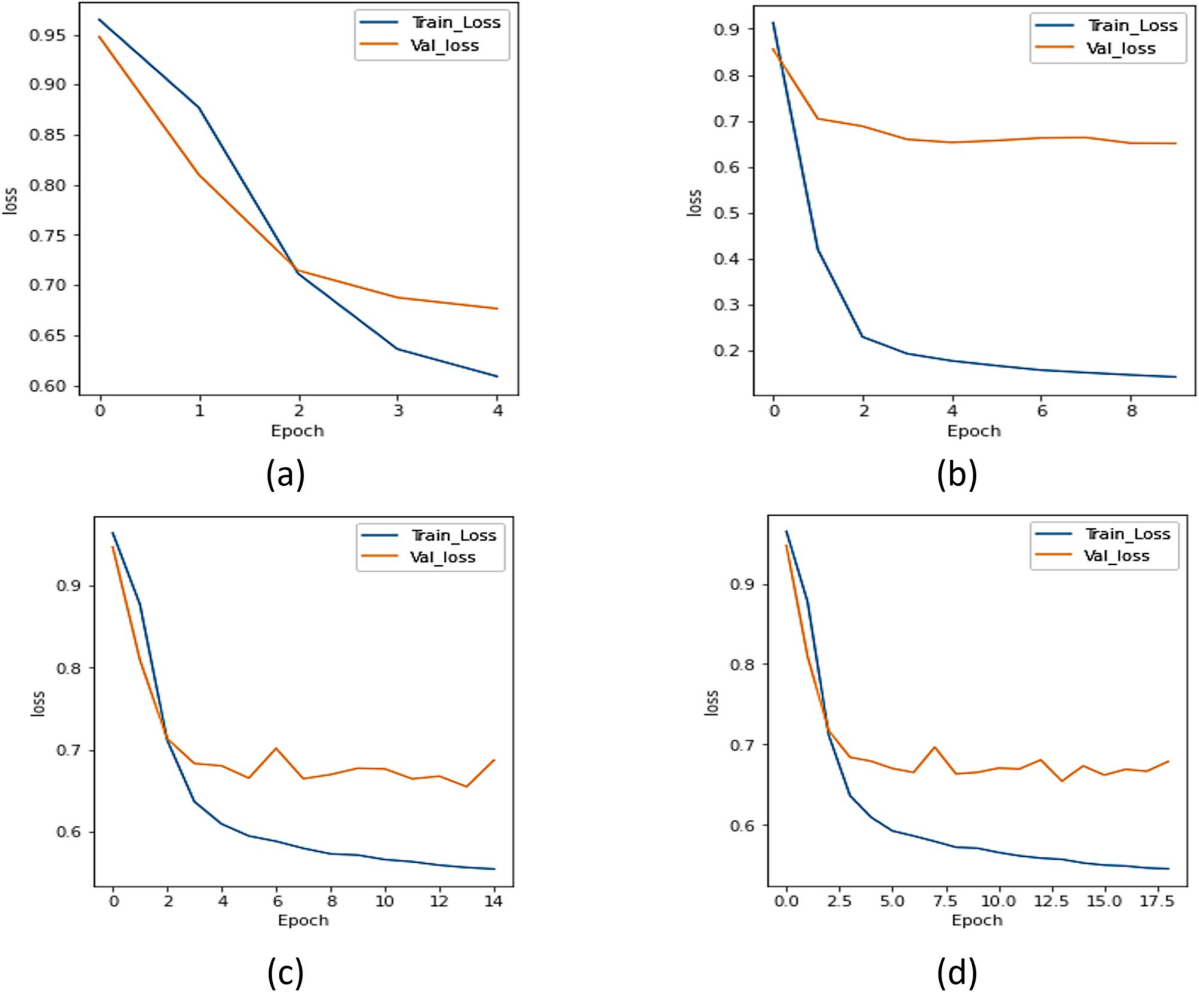
Loss analysis for different encoders using SGD optimizer. (a) Xception, (b) ResNet 101, (c) NASNet Mobile, and (d) UMobileNet V2 Model.

To compare the ensemble UMobileNet V2 model with other transfer learning models, model loss, dice coefficient, and IoU coefficient were considered. Fig 14A depicts the xception model’s loss curve, Fig 14B the ResNet 101 model’s loss curve, Fig 14C the NASNet model’s loss curve, and Fig 14D the results of the UMobileNet V2 model’s use of the SGD optimizer. Fig 14 shows that the graphs for Xception and ResNet 101 are comparable. The UMobileNet V2 model’s curves are identical to the other two models, NASNet Mobile. The model that is presented has the smallest loss value.

#### b.  Dice coefficient analysis

**Fig 15:**
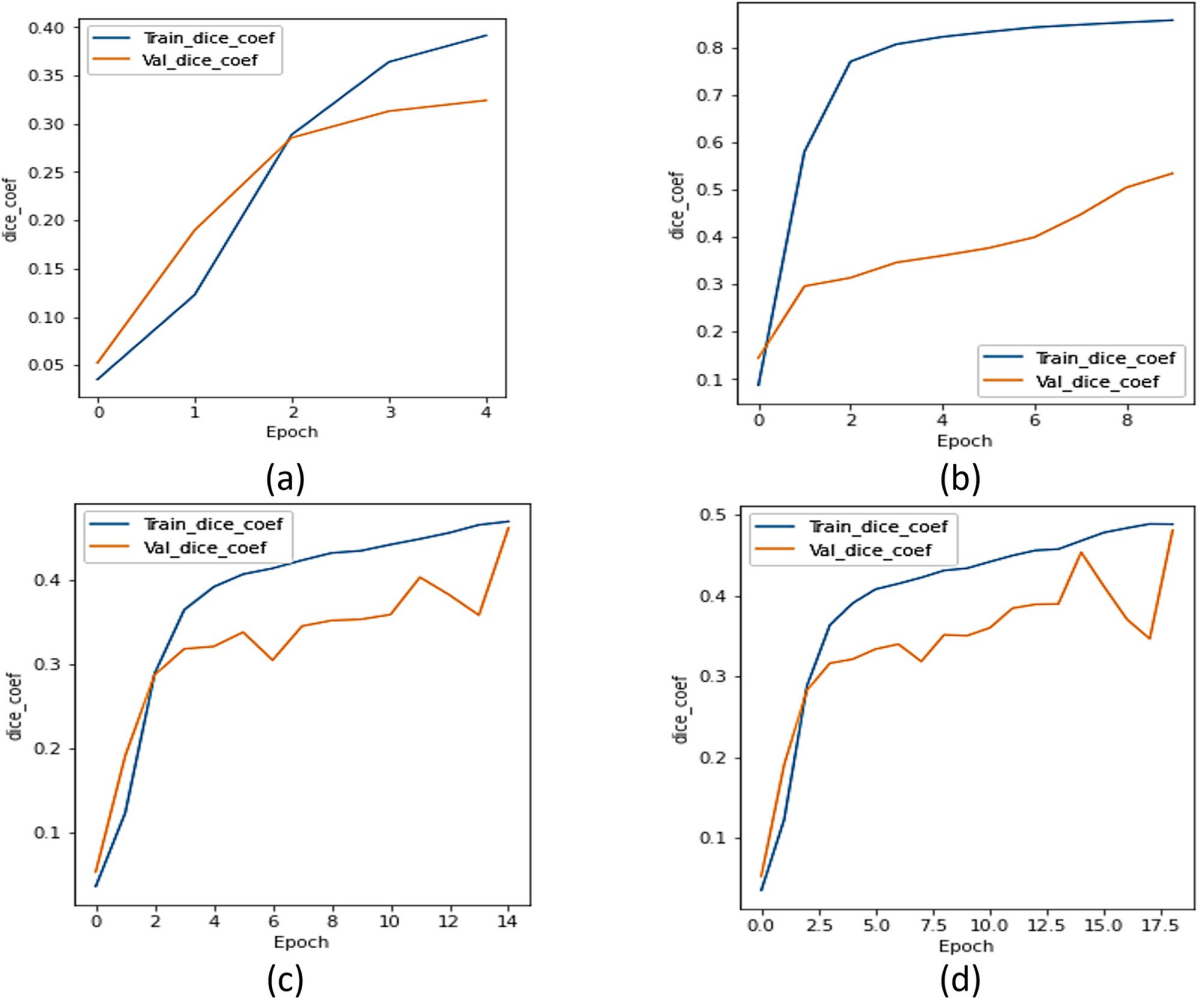
Dice coefficient analysis for different encoders using SGD optimizer. (a) Xception, (b) ResNet 101, (c) NASNet Mobile, and (d) UMobileNet V2 Model.

The UMobileNet V2 model and transfer learning models were assessed using the dice coefficient. Fig 15A depicts the dice coefficient curve for the xception model, Fig 15B the dice coefficient curve for the ResNet 101 model, Fig 15C the NASNet model, and Fig 15D the outcomes of the UMobileNet V2 model utilizing SGD optimizer. Fig 15 demonstrates that the UMobileNet V2 model provides the maximum value for the dice coefficient.

#### c.  IoU analysis

**Fig 16:**
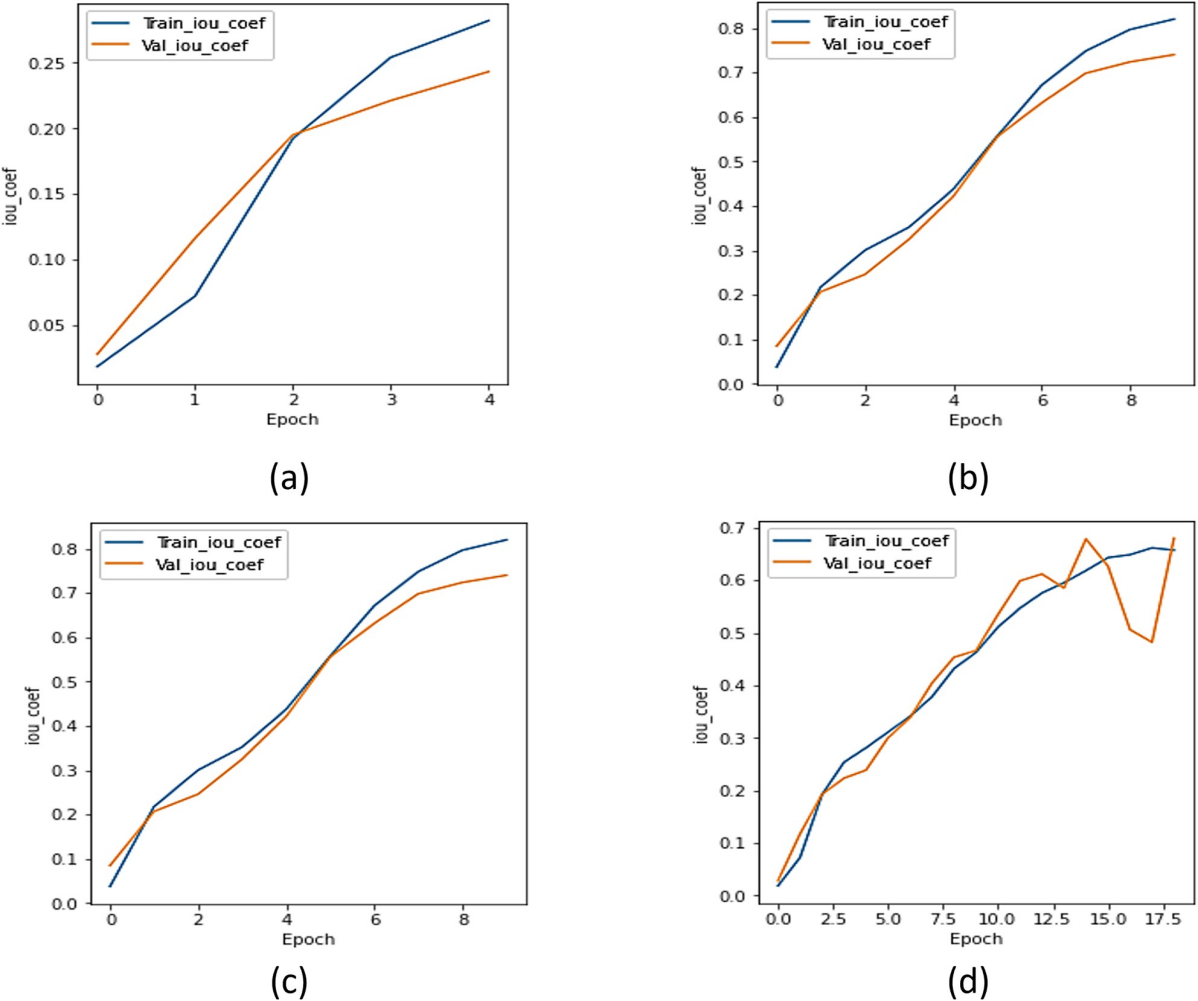
IoU analysis for different encoders using SGD optimizer. (a) Xception, (b) ResNet 101, (c) NASNet Mobile, and (d) UMobileNet V2 Model.

The IoU coefficient was used to compare the UMobileNet V2 model to all other transfer learning models. Fig 16A depicts the IoU curve for the xception model; Fig 16B shows the IoU curve for the ResNet 101 model; Fig 16C illustrates the IoU curve for the NASNet model; and Fig 16D depicts the plot of the UMobileNet V2 model utilizing SGD optimizer. Fig 16 demonstrates how the UMobileNet V2 model might produce the highest value of IoU.

**Fig 17:**
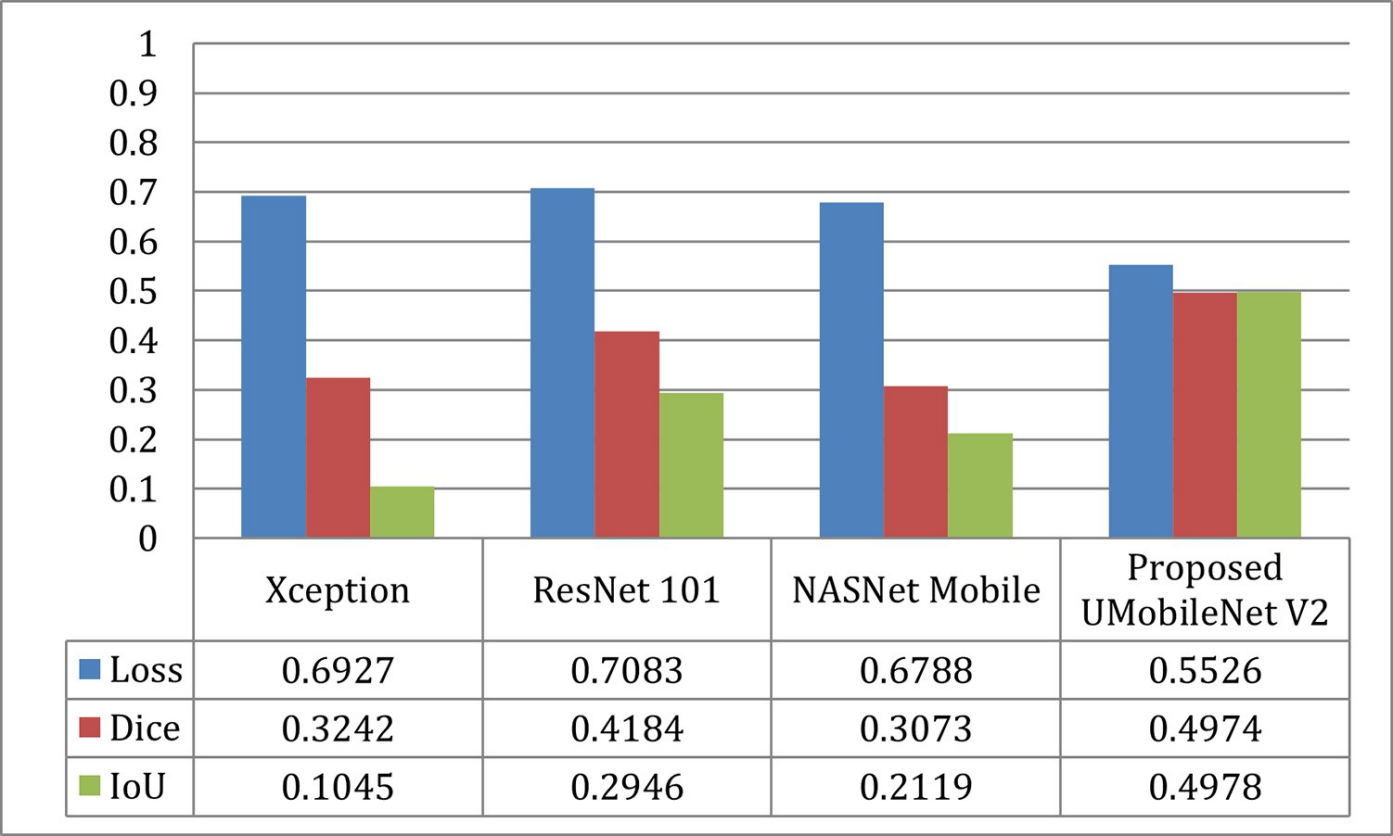
Results comparison of UMobileNet V2 model with different TL models at SGD optimizer using test dataset.

The results of the Adam optimizer for each model are contrasted in Fig 17 in terms of loss, dice coefficient, and IoU. As can be seen in the graphic, the recommended model outperformed earlier transfer learning models. The recommended model generated the highest dice coefficient, lowest loss value, and best IoU value when using the Adam optimizer.

### D.  Comparison of adam, RMS, and SGD optimizers

A fair comparison of the optimizers’ performance can be represented in graphs. This section shows the loss, dice, and IoU comparison graphs for all the models using Adam, RMS, and SGD optimizers.

#### a).  Loss comparison

**Fig 18:**
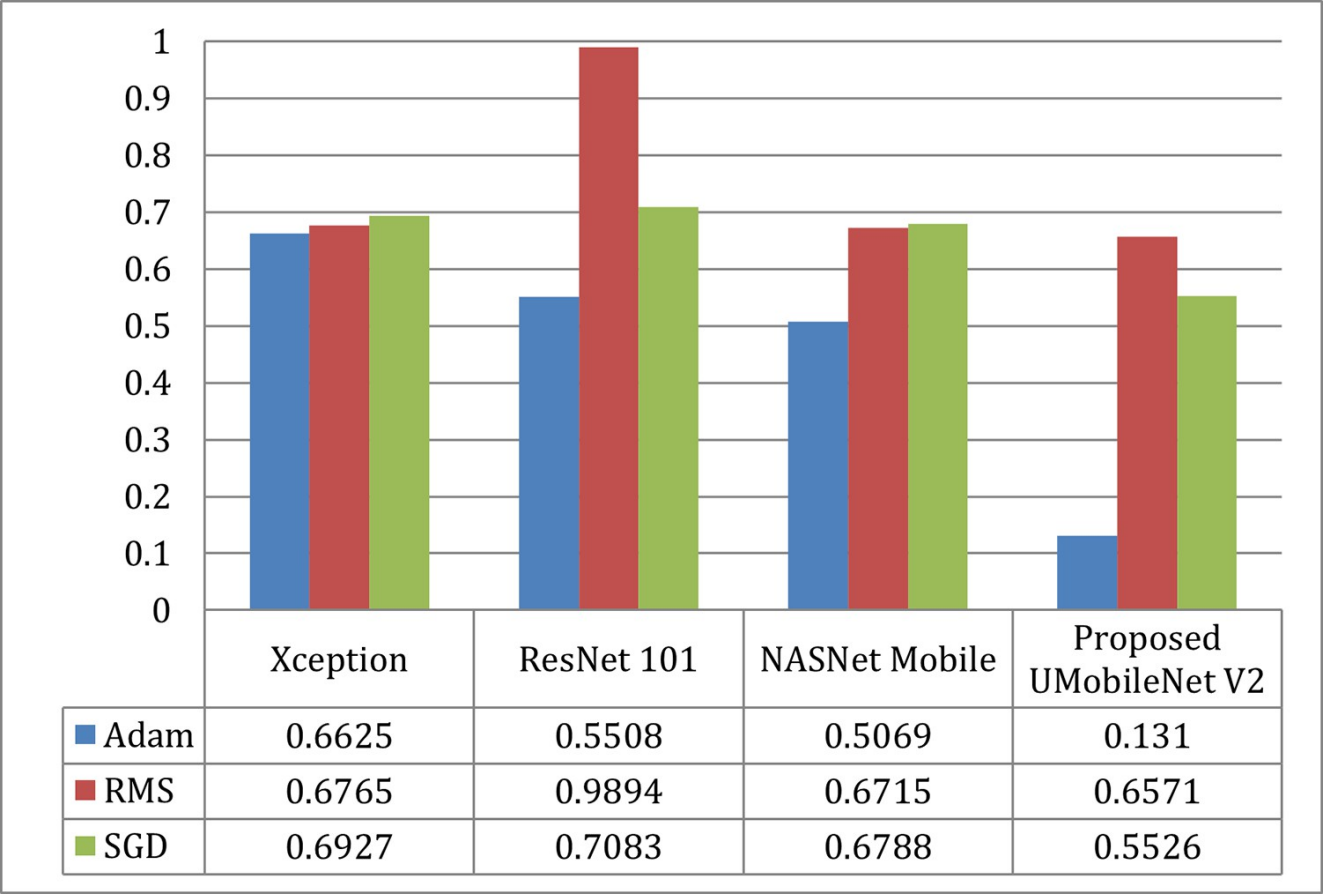
Loss comparison of UMobileNet V2 model with different encoders with different optimizers using test dataset.

The graph of loss comparison for all encoders and the UMobileNet V2 model employing Adam, RMS, and SGD optimizers is shown in Fig 18. Fig 18 demonstrates that, when compared to the other two optimizers, Adam’s performance is the best. In terms of models, the ensemble UMobileNet V2 outperforms the other three transfer learning models with the lowest loss values.

#### b).  Dice coefficient comparison

**Fig 19:**
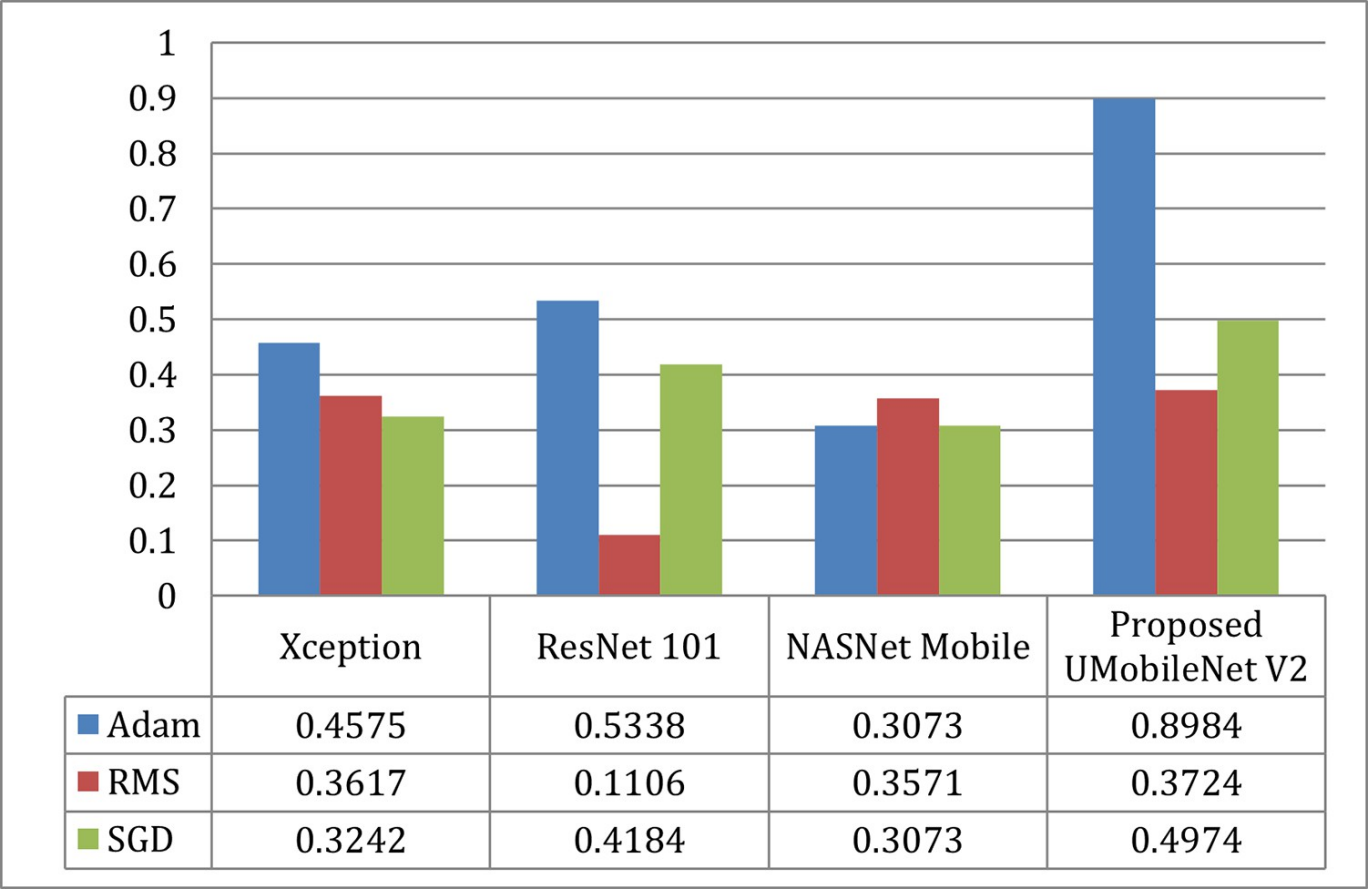
Dice coefficient comparison of UMobileNet V2 model with different encoders with different optimizers using test dataset.

Fig 19 compared the Dice Coefficients utilizing Adam, RMS, and SGD optimizers for all encoders and the UMobileNet V2 model. Fig 19 demonstrates that compared to the other two optimizers, Adam performs the best. In terms of models, the ensemble UMobileNet V2 outperforms the other three transfer learning models with the most significant dice coefficient values.

#### c).  IoU comparison

**Fig 20:**
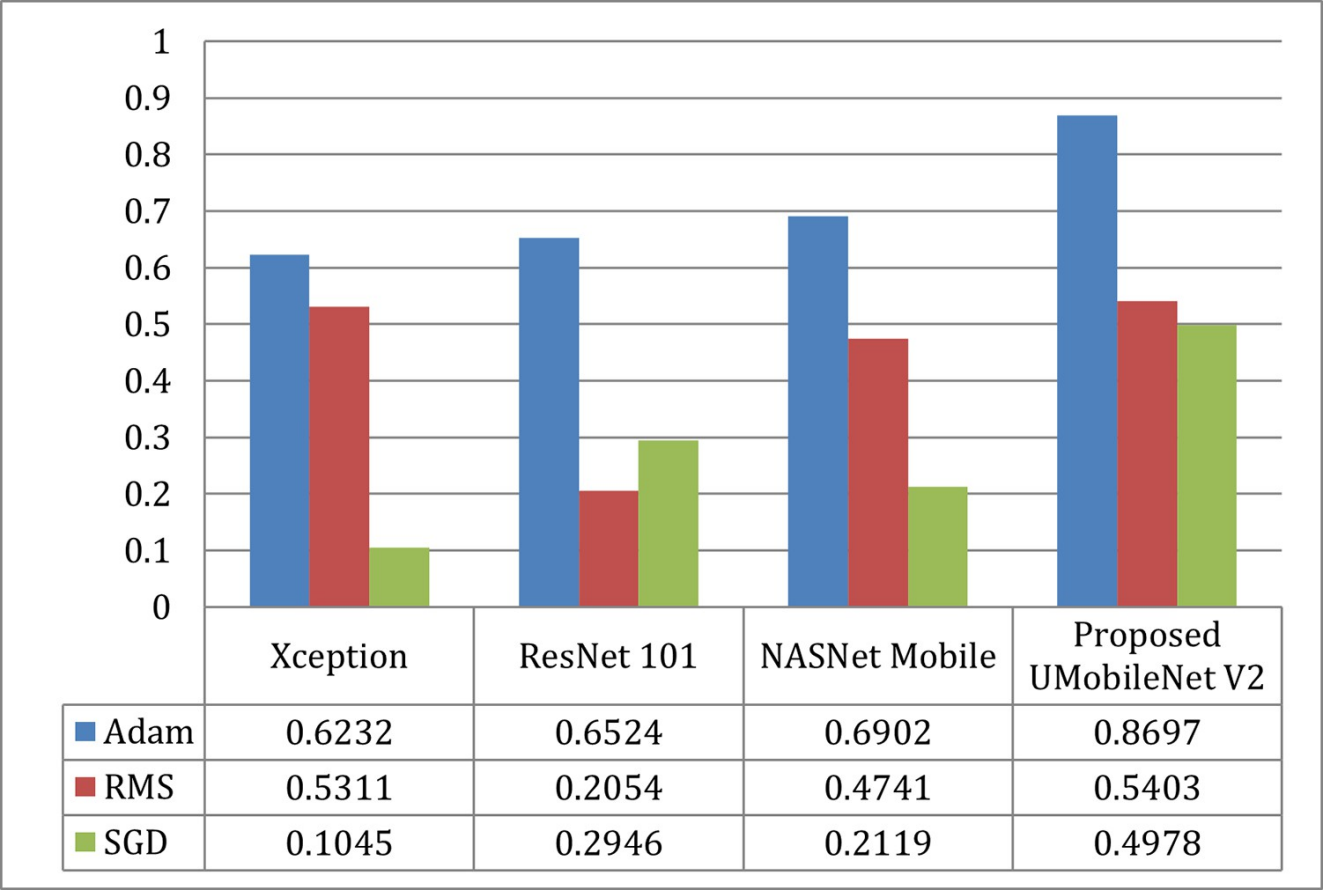
IoU comparison of UMobileNet V2 model with different encoders with different optimizers using test dataset.

The graph of IoU comparison for all transfer learning and UMobileNet V2 models utilizing Adam, RMS, and SGD optimizers is shown in Fig 20. Fig 20 demonstrates that Adam outperforms the other two optimizers regarding performance. In terms of models, the ensemble UMobileNet V2 outperforms the other three transfer learning models with the most significant IoU values.

From all of the comparisons and results, it is clear that the ensemble UMobileNet V2 outperforms existing transfer learning models like Xception, ResNet 101, and NASNet Mobile regarding all performance metrics, including loss, dice coefficient, and IoU. The Adam optimizer was used to get the final results. Compared to RMS and SGD optimizers, the performance of the Adam optimizer is the best. The final results are dice coefficient 0.8984, IoU value 0.8697, and model loss 0.1310.

### E.  Analysis of UMobileNet V2 model

**Fig 21:**
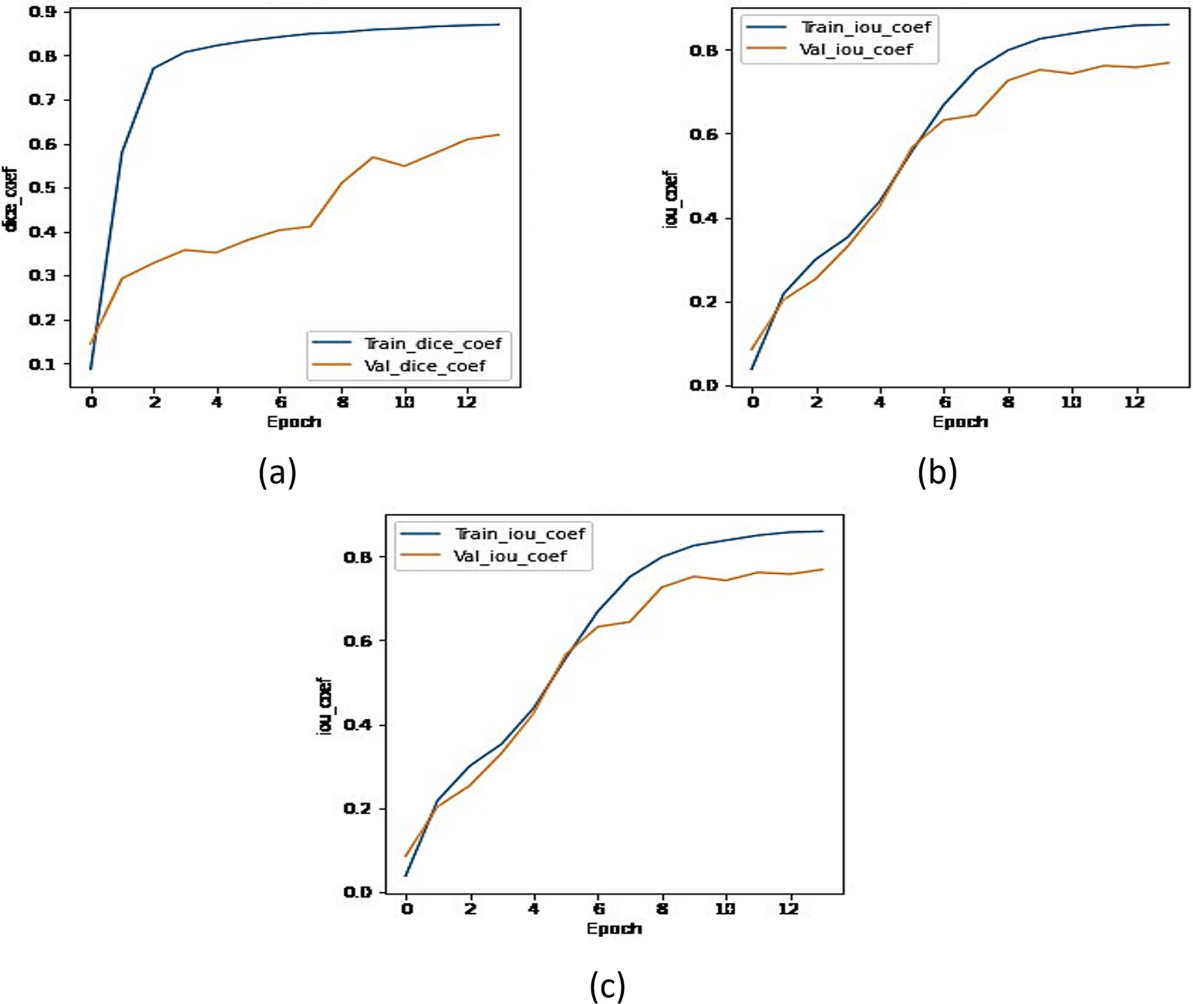
Graphs of the Optimized Model (a) Dice Coefficient, (b) IoU Coefficient, and (c) Loss.

This section shows the analysis of the UMobileNet V2 with Adam optimizer. Fig 21 shows the graph obtained by running the model with more epochs to check the performance of UMobileNet V2 model at higher number of epochs. The graphs show that the results are not much improved for higher epochs. The graphs show rising dice and IoU and decreasing values for loss for the initial epochs, getting plateau for higher epochs showing its saturation.

**Fig 22:**
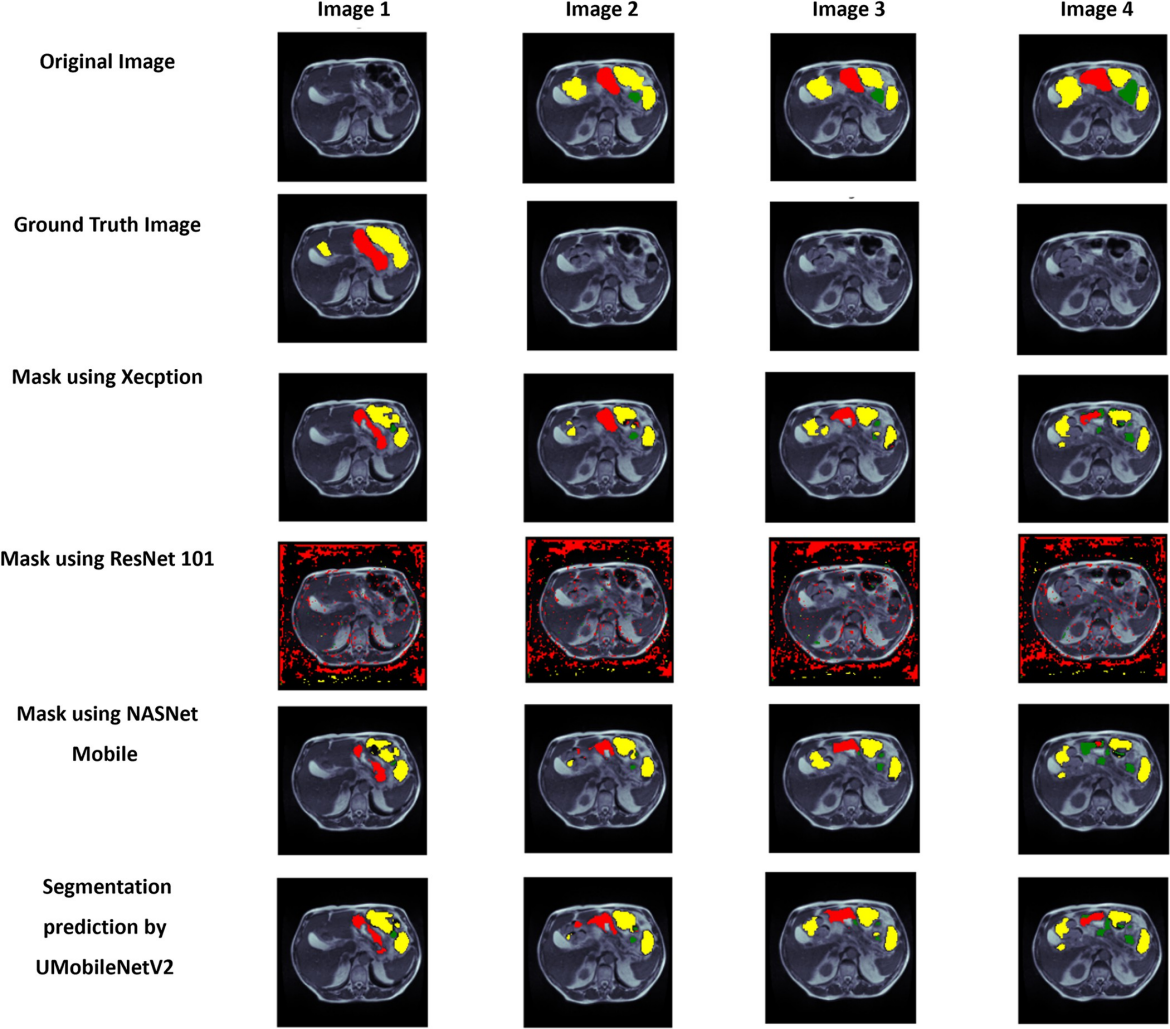
Visual examination of the outcomes in the form of pictures. Here, yellow is used to show the big intestine, green is used to represent the small intestine, and red is used to represent the stomach.

The visual analysis of the UMobileNet V2 model is shown in Fig 22. The output images from the UMobileNet V2 and other transfer learning models are displayed in Fig 22. The image comprised the input image, their mask, and each model’s predicted images. Here, yellow indicates the large intestine, green represents the small intestine, and red represents the stomach. Fig 22 demonstrates how closely the UMobileNet V2 model’s output images resemble the input images. Regarding visual analysis, UMobileNet V2 model outperforms other encoders.

Table 4 presents a comparative analysis of three different optimizations—Adam, RMSprop, and SGD—applied to the UMobileNet V2 model for segmentation. The evaluation metrics include the Dice Coefficient, measuring the overlap between predicted and true segmentation masks; the IoU Coefficient, quantifying the intersection over the union of the same masks; and the Loss, reflecting the model’s performance in minimizing the discrepancy between predictions and ground truth. Adam demonstrates superior performance with a Dice Coefficient of 0.8984 and an IoU Coefficient of 0.8697, indicating robust segmentation accuracy. In contrast, RMSprop and SGD exhibit lower scores across these metrics. The Loss metric, representing the overall model performance, is lowest for Adam and highest for RMSprop, further emphasizing Adam’s efficacy in this context. Additionally, the table includes the Time Spent column, illustrating the computational time required for each optimization algorithm—Adam, RMSprop, and SGD—to train the model. The presented information provides valuable insights into the trade-offs between optimization algorithms in terms of both segmentation accuracy and computational efficiency.

**Table 4:** Analysis of optimized model based on different epochs.

	Dice Coefficeint	IoU Coefficient	Loss	Processing Time
**Adam**	0.8984	0.8697	0.1310	2 hrs 34 min
**RMS**	0.3724	0.5403	0.6571	2 hrs 57 min
**SGD**	0.4978	0.4978	0.5526	3 hrs 15 min

### F.  Comparison of UMobileNet V2 model with other segmentation models

The Table 5 presents performance metrics for various segmentation models including FPN, UNet, PSPNet, and UMobileNet V2. Each model is evaluated based on three parameters Dice Coefficient, IoU Coefficient, and Loss. The Dice Coefficient measures the overlap between the predicted and ground truth segmentation masks, with UMobileNet V2 achieving the highest score of 0.8984, indicating superior segmentation accuracy. Similarly, UMobileNet V2 also achieves the highest IoU Coefficient of 0.8697, again suggesting better overall performance in capturing the intersection between predicted and true segmentation areas. In terms of Loss, UMobileNet V2 has the lowest value of 0.1310, indicating the model’s ability to minimize errors during training. Overall, UMobileNet V2 demonstrates strong performance across all metrics compared to the other segmentation models listed in the Table 5.

**Table 5:** Comparison of UMobileNet V2 model with other segmentation models.

Segmentation Model	Dice Coefficient	IoU Coefficient	Loss
**FPN**	0.8675	0.8332	0.1451
**UNet**	0.8554	0.8319	0.1520
**PSPNet**	0.8642	0.8431	0.1565
**UMobileNet V2**	0.8984	0.8697	0.1310

## 5. State-of-the-art comparison

Table 6 provides a complete overview of recent studies focusing on semantic segmentation of gastrointestinal structures in the UW Madison dataset, highlighting the Dice coefficient as an evaluation metric. Various techniques have been employed, including UNet with an attention mechanism [43], Levit-UNet++ [44], a combination of UNet and Mask RCNN [45], Multiview UNet [46], and an Ensemble Model [47], each achieving different levels of segmentation accuracy with Dice values ranging from 0.36 to 0.88. More recent approaches in 2023 include FPN+Efficient Net B0 [47] with a Dice coefficient of 0.8975, UNet model [48] with a Dice coefficient of 0.8854, and PSPNet+ResNet 34 [49] with a Dice coefficient of 0.8842. The UMobileNet V2 Model, featuring a MobileNetV2 encoder embedded within a UNet architecture, outperforms previous methods with a Dice coefficient of 0.8984, demonstrating promising results in segmenting gastrointestinal structures in the specified dataset.

**Table 6:** State-of-the-art comparison on UW Madison GI tract dataset.

Ref	Year	Method	Dice
[42]	2022	UNet with an attention mechanism	0.78
[43]	2022	Levit-UNet++:	0.79
[44]	2022	Combination of UNet and Mask RCNN	0.51
[45]	2022	Multiview UNet	0.36
[46]	2022	Ensemble Model	0.88
[47]	2023	FPN+Efficient Net B0	0.8975
[50]	2023	UNet	0.8854
[48]	2023	PSPNet+ResNet34	0.8842
**UMobileNet V2 Model**		**MobileNetV2 encoder embedded with UNet**	**0.8984**

## 6. Conclusion and future scope

The gastrointestinal system is significant part of the human body since it controls digestion and absorption of food. GI cancer numbers have been steadily rising in recent years. The most frequent treatment for GI cancer is radiation treatment, which includes directing X-rays toward the tumor while avoiding healthy organs. So, there is a need for a system that can automatically partition the GI organs to protect healthy organs from X-ray beams and speed up cancer therapy. This paper uses the UW Madison dataset to present a UMobileNetV2 model for segmenting GI organs like the small intestine, large intestine, and stomach. The UMobileNet V2 model uses UNet architecture with MobileNetV2 transfer learning model as an encoder to downsample the feature for semantic segmentation. The results of UMobileNet V2 model were compared with three transfer learning models: Xception, ResNet 101, and NASNet mobile with loss, dice, and IoU. The UMobileNet model, combined with the Adam optimizer, surpasses all encoders, achieving values of 0.8984 for the dice coefficient, 0.8697 for the IOU, and 0.1310 for the validation loss.

While UNet is renowned for its efficiency, U-Net is designed with a narrowing path (encoder) and wide path (decoder). However, the context information available to each pixel in the decoding path is limited to the corresponding region in the encoding path. This limitation can affect the capability of model to capture long-range dependencies and global context information. In the future, the combination of different decoder models with the combination of MobileNet V2 as encoder can be analyzed for the GI tract semantic segmentation.
